# Multidisciplinary Perspectives of Challenges in Infective Endocarditis Complicated by Septic Embolic-Induced Acute Myocardial Infarction

**DOI:** 10.3390/antibiotics13060513

**Published:** 2024-05-31

**Authors:** Elena Stamate, Oana Roxana Ciobotaru, Manuela Arbune, Alin Ionut Piraianu, Oana Monica Duca, Ana Fulga, Iuliu Fulga, Alexia Anastasia Stefania Balta, Adrian George Dumitrascu, Octavian Catalin Ciobotaru

**Affiliations:** 1 Department of Cardiology, University Emergency Hospital of Bucharest, 169 Splaiul Independentei St., 050098 Bucharest, Romania; elena.stamate94@yahoo.com; 2 Faculty of Medicine and Pharmacy, Dunarea de Jos University of Galati, 35 AI Cuza St., 800010 Galati, Romania; roxana_hag@yahoo.com (O.R.C.); oanam.duca@gmail.com (O.M.D.); ana.fulgaa@yahoo.com (A.F.); fulgaiuliu@yahoo.com (I.F.); alexia.balta@ugal.ro (A.A.S.B.); coctavian72@gmail.com (O.C.C.); 3 Railway Hospital Galati, 6 Alexandru Moruzzi St., 800223 Galati, Romania; 4 Infectious Diseases Clinic Hospital “Sf. Cuv. Parascheva”, 393 Traian St., 800179 Galati, Romania; 5 Emergency County Hospital Braila, 2 Sos. Buzaului St., 810325 Braila, Romania; 6 Saint Apostle Andrew Emergency County Clinical Hospital, 177 Brailei St., 800578 Galati, Romania; 7 Division of Hospital Internal Medicine, Department of Medicine, Mayo Clinic Florida, 4500 San Pablo Rd S, Jacksonville, FL 32224, USA; dumitrascu.adrian@mayo.edu

**Keywords:** coronary, septic embolism, endocarditis, myocardial infarction, antibiotic therapy

## Abstract

Background: Infective endocarditis (IE) management is challenging, usually requiring multidisciplinary collaboration from cardiologists, infectious disease specialists, interventional cardiologists, and cardiovascular surgeons, as more than half of the cases will require surgical procedures. Therefore, it is essential for all healthcare providers involved in managing IE to understand the disease’s characteristics, potential complications, and treatment options. While systemic embolization is one of the most frequent complications of IE, the coronary localization of emboli causing acute myocardial infarction (AMI) is less common, with an incidence ranging from 1% to 10% of cases, but it has a much higher rate of morbidity and mortality. There are no guidelines for this type of AMI management in IE. Methods: This narrative review summarizes the current knowledge regarding septic coronary embolization in patients with IE. Additionally, this paper highlights the diagnosis and management challenges in such cases, particularly due to the lack of protocols or consensus in the field. Results: Data extracted from case reports indicate that septic coronary embolization often occurs within the first two weeks of the disease. The aortic valve is most commonly involved with vegetation, and the occluded vessel is frequently the left anterior descending artery. Broad-spectrum antibiotic therapy followed by targeted antibiotic therapy for infection control is essential, and surgical treatment offers promising results through surgical embolectomy, concomitant with valve replacement or aspiration thrombectomy, with or without subsequent stent insertion. Thrombolytics are to be avoided due to the increased risk of bleeding. Conclusions: All these aspects should constitute future lines of research, allowing the integration of all current knowledge from multidisciplinary team studies on larger patient cohorts and, subsequently, creating a consensus for assessing the risk and guiding the management of this potentially fatal complication.

## 1. Introduction

Infective endocarditis (IE) is an inflammatory heart disease caused by microbial invasion of the heart valves and endocardium. Despite improvements in the management of IE, the mortality rate at discharge remains high, ranging between 20 and 30% [1]. Mortality and morbidity of IE are mainly due to the progression to uncontrolled infection (UI) and embolic events (EE) and are also due to heart failure (HF) [2]. The septic embolic event is a common complication in IE [3] and is associated with a poor prognosis. Left-side heart valves’ infection often has the highest embolic risk [4]. Surgical intervention is highly controversial for septic EE prevention, whereas surgery is well-established for HF and UI management [5]. As a result, there is no consistent indication for surgery to prevent septic embolism according to the AHA and ESC (American Heart Association and European Society of Cardiology) [6].

According to large registries, septic embolic events affect about 20–40% of patients with IE [3]. In many cases, at the time of diagnosis of IE, septic embolic events are already present. Septic embolic events from IE are the migration of fragments of infectious material from the IE vegetation into the pulmonary or systemic circulation at any point. Current data suggest that the most common organ affected by septic embolization is the brain, accounting for about 50% of cases, followed by the spleen, the pulmonary system [7,8], the lungs, the kidneys, and the extremities, with a variable incidence [8]. This variable index is given by the extent to which asymptomatic EE is included or not included in the calculation [7,8].

Because it causes vascular occlusion, a septic embolism can lead to various degrees of ischemia in the area supplied by the occluded vessel. Most often, systemic embolization occurs in IE, affecting the valves on the left side of the heart, and can lead to stroke, splenic infarction, renal infarction, limb ischemia, or acute myocardial infarction if septic emboli occlude coronary arteries [9]. Less common in terms of IE complications, but increasingly recognized, acute myocardial infarction (AMI) has a variable incidence, between 1 and 10%. Still, it has a high mortality rate at discharge of up to 64% in the AMI population compared to the population without AMI [10]. A higher risk of embolization is associated with the following factors: length of vegetation > 10 mm, mobile vegetation, infection with non-viridans streptococci and staphylococci, and the presence of a previous embolism [11,12]. According to the 2023 ESC recommendations, one of the main indications for surgery to prevent septic embolism is the presence of persistent vegetation larger than 10 mm despite antibiotic therapy. Even when accounting for all surgical risks, surgery in these patients can still increase survival by up to 20% in one year, according to the 2023 ESC Infective Endocarditis Guidelines [13]. Transthoracic cardiac ultrasound (TTE) and transesophageal cardiac ultrasound (TOE) are essential imaging methods for the diagnosis, management, and monitoring of IE. Clinical suspicion of IE should be investigated initially by TTE. However, as TTE tends to have a variable sensitivity for IE diagnosis, when clinical suspicion persists, it is necessary to perform TOE to confirm or deny the diagnosis [14].

A rare embolic phenomenon of IE is the mycotic aneurysm. It is an aneurysmal dilatation of the blood vessel, caused by the infection of the vessel wall following hematogenous insemination from a septic embolism [11]. Mycotic aneurysms most commonly occur in the cerebral arteries, visceral arteries, and aortic artery. Embolic myocardial infarction is a less common complication of IE, but one that is becoming increasingly recognized. Clinical suspicion is extremely important, especially in patients with AMI and fever. Multidisciplinary management includes clinical cardiologists, infection disease specialists, cardiovascular surgeons, interventional cardiologists, anesthesia, intensive care physicians, and other related specialties such as general surgeons or vascular surgeons. The heart team’s training is essential in choosing the best treatment option for the patient. The management of this condition is essential and must be aggressive from the start. This consists of antibiotic therapy first and foremost, but also early cardiac surgery to remove the source of infection [11,13,15,16].

When considering intervention for septic embolic events in IE which is complicated with AMI, the use of primary percutaneous coronary intervention (PCI) appears to be the most viable solution. Stent implantation may be performed, but the risk of developing a mycotic aneurysm must be considered. However, evidence and efficacy are limited for this approach. It is considered that balloon inflation may cause mobility of the vegetation and subsequently bring an additional risk of embolic phenomena. Thrombolytics are not a viable solution either, as they seem to present complications such as embolic risks and significant bleeding [15]. They may be useful in a few extreme cases, such as in cases of septic pulmonary embolism in IE, where thrombolytic therapy has been superior to other anticoagulant therapies [16].

The 2017 ESC Clinical Practice Guidelines do not routinely recommend thrombaspiration during primary percutaneous coronary intervention in septic embolization. Mechanical thrombus aspiration to remove the infected material may be considered, but there are not enough data available to support this approach [17]. The most important recommendation from high-quality studies remains the use of antibiotics for the treatment of IE [13,14].

In this narrative review of the literature, the authors aim to assess the status of infective endocarditis complicated by acute myocardial infarction induced by septic embolism, highlighting the latest advancements in diagnosis and management. Although considered to be a rare complication, it is becoming more prevalent due to the evolution of diagnostic and management methods. However, physicians are facing many challenges when treating this type of acute myocardial infarction. These challenges pertain to the lack of guidelines/standardization of its management or the lack of treatment outcomes, as only case reports or small studies addressing this topic have been published so far. These challenges represent future lines of research in this field that would contribute to the evolution of patient-based personalized medicine.

Our article is of particular importance for medical research, since, as mentioned in a 2021 article, there are no more than 100 described cases with septic embolization in the coronary arteries from infective endocarditis complicated by acute coronary syndrome [18].

## 2. Literature Review

### 2.1. Methodology

We conducted a comprehensive review of the current literature, including original articles which addressed the issue of septic embolization in IE complicated with acute myocardial infarction. We performed extensive searches on the PubMed, Google Scholar, ScienceDirect, Elsevier, Scopus, Web of Science, and Cochrane databases to identify relevant manuscripts. We used three sets of keywords to recognize terms from the title, abstract, and keywords of the studies: (i) The first set of keywords included terms associated with infective endocarditis, such as “embolization”, “coronary artery”, “endocarditis”, “diagnosis”, “treatment”, and “acute myocardial infarction” or “acute coronary syndrome”. However, studies using these methodologies are likely to incorporate terms such as “infective endocarditis” or “acute myocardial infarction” in their abstracts or keywords. (ii) The second set of keywords included complications following septic embolization from infective endocarditis, such as “embolic events”, “septic coronary embolism”, “septic shock”, “cardiogenic shock”, and “heart failure”. Thus, compound searches were performed using the terms “acute myocardial infarction” or “acute coronary syndrome” combined with “infective endocarditis” and “septic coronary embolism”. We restricted our search to papers published in English, and we found more than 119 relevant manuscripts.

We removed duplicate articles and then conducted a detailed evaluation of the abstracts and titles to determine their suitability for inclusion. The selection criteria focused on studies and case reports that addressed infective endocarditis complicated with septic embolization in coronary arteries. Subsequently, we systematically applied the selection criteria to evaluate the studies. The studies were assessed based on the following criteria: (1) journal, (2) publication date, (3) study design/case report design, (4) results, and (5) conclusions. We eliminated the studies not written in English. To ensure data quality, we paid close attention to specific aspects of the studies meeting the inclusion criteria, such as justification, method design, results, discussions, and conclusions. We excluded the studies with methodological bias, skewed results, or data interpretation that could negatively impact the outcome.

The exclusion criteria were (a) articles in languages other than English, (b) retracted studies, and (c) articles about IE that did not have septic embolization as a complication.

### 2.2. Results

**Figure 1:**
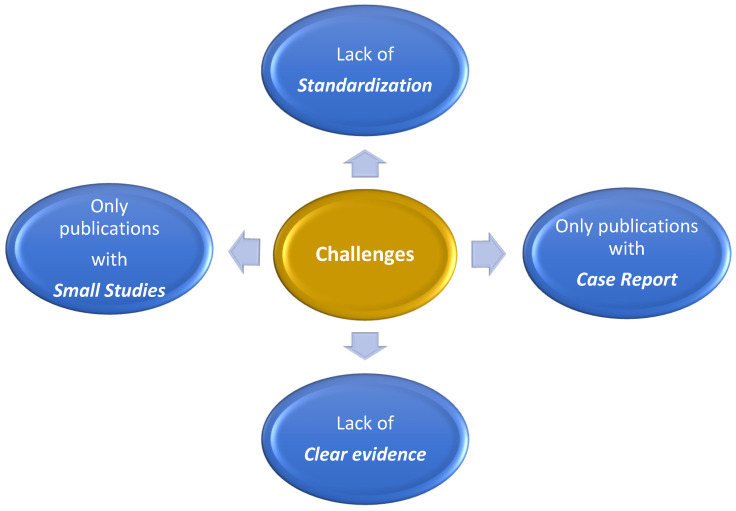
Challenges in infective endocarditis complicated by septic embolic-induced acute myocardial infarction.

**Figure 2:**
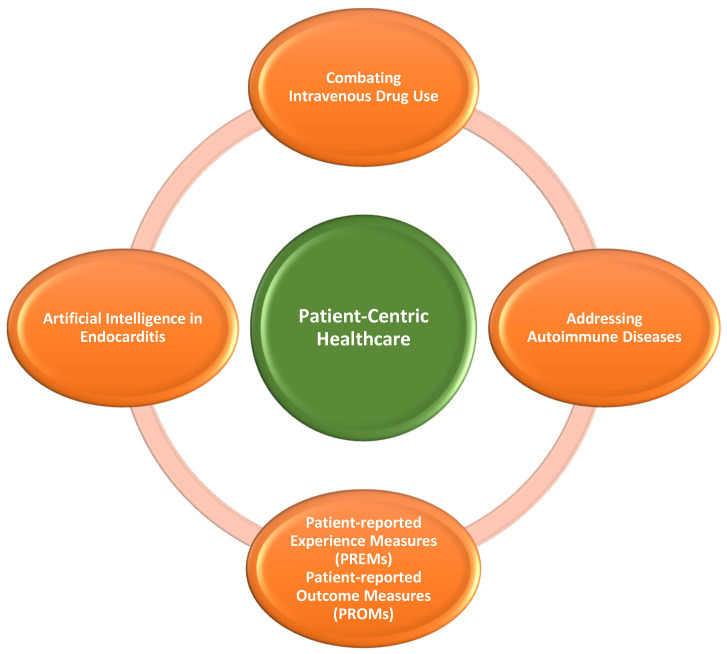
Future directions in combating the burden of endocarditis.

After a thorough review and assessment of 74 articles, we identified and included a subset of 31 papers that were directly relevant to our research (see Table 1). These selected studies provided valuable insights into the diagnosis and management of IE-associated septic embolism causing AMI, presenting current treatment methods in these cases, together with their challenges. These challenges are summarized schematically in Figure 1. The future directions that require additional work to reduce the burden of this infectious disease are listed in Figure 2.

#### 2.2.1. Main Points Regarding Acute Myocardial Infarction Induced by Septic Embolism from Infective Endocarditis

To make the reader’s work easier, we present the analysis of the data from the 31 articles:

- The most frequently affected valve

**Figure 3:**
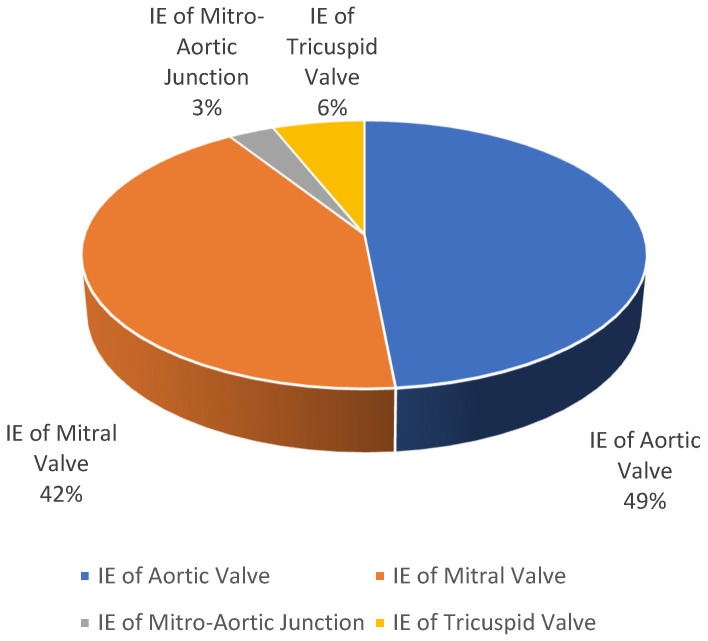
Valves involved in infective endocarditis complicated by septic embolic-induced acute myocardial infarction.

We reviewed 31 original articles describing 31 clinical cases. We observed that the most involved valve was the aortic valve (native and prosthetic), with a total of 16 cases (49%), followed by the mitral valve with a total of 14 cases (42%), the tricuspid valve with 2 cases (6%), and the mitral–aortic junction with 1 case (3%), as summarized in Figure 3. Among all the articles analyzed, four articles documented cases of infective endocarditis on multiple valves, as follows: the mitral valve and aortic valve were both affected and documented in three cases, and the mitral valve and the tricuspid valve were both affected and documented in one case.

- The type of AMI determined by septic embolization

**Figure 4:**
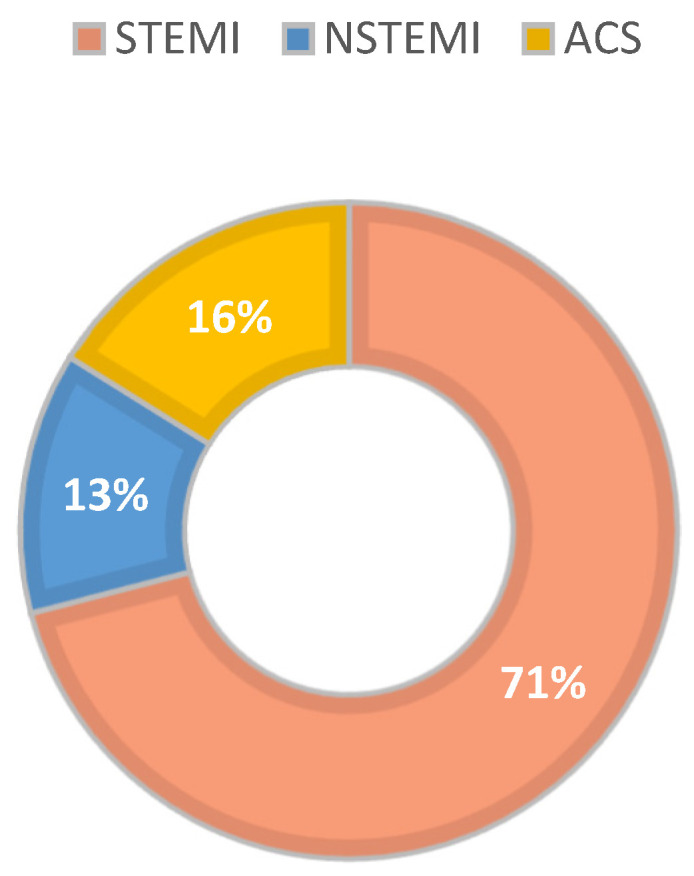
Infective endocarditis complicated by different types of septic embolic-induced acute myocardial infarction.

IE complicated by septic embolism in the coronary arteries caused different types of AMI: ST elevation myocardial infarction (STEMI) in the majority of cases, i.e., 22 cases (71%), acute coronary syndrome (ACS) in 5 cases (16%), and NON-ST elevation myocardial infarction (NSTEMI) in 4 cases (13%), as we have illustrated in Figure 4.

- The pathogens involved in endocarditis

**Figure 5:**
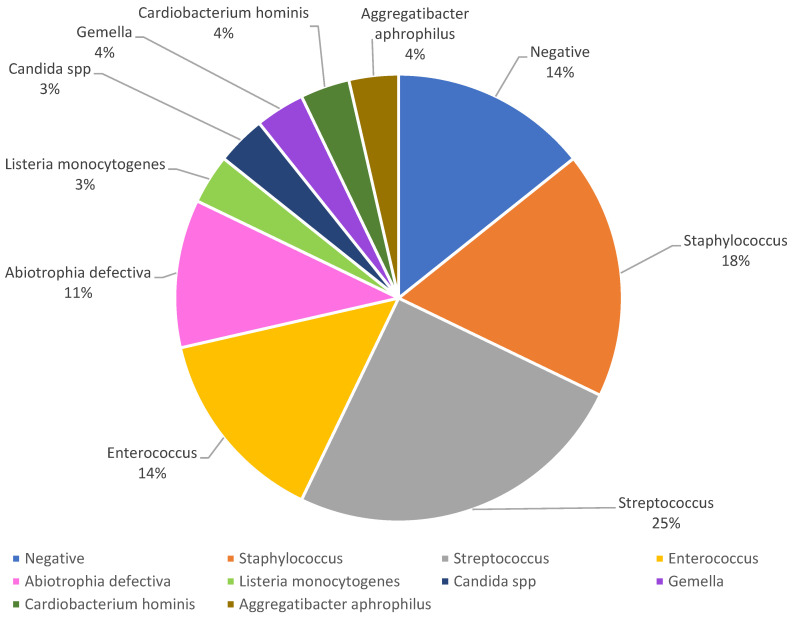
Culture results in patients with infective endocarditis complicated by septic embolic-induced acute myocardial infarction.

The pathogens were primarily isolated from blood cultures or from the septic embolus culture retrieved from the coronary artery in one case in which the patient had already undergone intravenous antibiotic treatment before their presentation to the hospital. The culture isolates’ results are summarized in Figure 5, as follows: negative blood cultures in four cases (14%), *Streptococcus* spp. in seven cases (25%), *Staphylococcus* spp. in five cases (18%), *Enterococcus* spp. in four cases (14%), *Abiotrophia defectiva* in three cases (11%), one case with *Listeria monocytogenes* (3.2%), one case with *Candida* spp. (3.2%), one case with *Gemella* (3.2%), and one case each with bacteria classified in the HACEK group—*Aggregatibacter aphrophilus* (4%) and *Cardiobacterium hominis* (4%). Out of the ones with negative hemocultures, one case was classified as Libman–Sacks endocarditis.

#### 2.2.2. Exploring Septic Embolization in Endocarditis: Focus on Acute Myocardial Infarction

- Infective Aortic Valve Endocarditis Complicated with Septic Coronary Artery Embolization and AMI
  1. IE of the Aortic Valvular Prosthesis

It is recognized that the most severe form of endocarditis is infective prosthetic valve endocarditis [11]. Inevitably, patients with significant valvular heart disease eventually undergo either the replacement of the affected valve or only valve repair. Valve repair is frequently performed, especially for mitral and tricuspid regurgitation, but valve replacement also remains common, especially in adult patients. According to the 2024 Guidelines for the Evaluation of Prosthetic Valve Function with Cardiovascular Imaging, diagnostic imaging methods are often required to assess the function of the valvular prosthesis. Out of all imaging methods, echocardiography is a non-invasive, first-line method for assessing prosthetic valve function. In selected situations, TTE evaluation is complemented by TOE for the further refinement of valve function and morphology. Cardiac magnetic resonance (MRI) and cardiac computed tomography (CT) have recently improved their role in cardiac valve evaluation [49].

Valencia J. et al. published in 2021 a case of a 69-year-old patient with endocarditis from a 1994 Medtronic–Hall (Medtronic, Minneapolis, MN, USA) 25 mm metal aortic valve prosthesis implanted, complicated by STEMI through septic embolization to the coronary arteries, and multiple images of cortical and subcortical ischemic infarcts, most likely of embolic origin as well. TTE was complemented by TOE, which revealed a suggestive image of 7 mm × 2 mm vegetation attached to the aortic valve on the ventricular side. The blood cultures collected from this patient were positive for *Staphylococcus aureus* and consistent with cultures isolated later from the vegetation. Treatment with empirical antibiotics with rifampicin, cloxacillin, meropenem, and daptomycin was initiated, but the clinical course was complicated with STEMI. Emergency percutaneous coronary angiography was performed, and an occlusion in the distal segment of the anterior descending artery (LAD) was noted, without other significant lesions; a thrombectomy was successfully performed. Thrombolysis was also administered. The recommended subsequent treatment was anticoagulation, as there were no atherosclerotic plaques to justify antiplatelet therapy. Subsequently, the patient underwent valve replacement surgery, and the mechanical valve was replaced with a biological valve, with a good postoperative outcome. Unfortunately, the patient required reoperation for sternal wound dehiscence and Pseudomonas aeruginosa infection, with an unfavorable outcome and death 5 months after their admission [18].

Another case of infective endocarditis of the bioprosthetic valve used to repair a bicuspid aortic valve was published by Cohen A. et al. The patient was a 61-year-old woman who suffered a septic embolic complication of the coronary arteries from IE with subsequent NSTEMI. TOE revealed several images suggestive of aortic valve vegetations, the largest measuring 1.7 × 1.0 cm at the level of the bioprosthetic aortic valve, as well as an image suggestive of an abscess. She received an empiric antibiotic treatment with vancomycin and ceftriaxone, pending the result of blood cultures. These were positive for *Streptococcus mitis*/*oralis*. For the acute myocardial infarction, coronary angiography was performed, which revealed 95% occlusion at the LAD level, highly suggestive of a septic embolism. The patient’s clinical course was complicated by cardiogenic shock, requiring norepinephrine and transfer to a center where she underwent emergency surgery. The aortic valve was replaced, coronary artery bypass grafting was performed, and the ventricular septal defect was closed following aortic root repair. Postoperatively, she developed *Pseudomonas pneumonia* infection and required additional antibiotic treatment with ciprofloxacin [19].

Denegri A. et al. published a case report of a 77-year-old man who presented to the hospital with fever and dyspnea three months after aortic valve replacement surgery with a biological prosthesis for symptomatic, severe aortic stenosis. This infective endocarditis of the biological aortic valve was complicated with STEMI due to coronary embolization and acute left upper-limb ischemia. Empirical antibiotic therapy with broad-spectrum antibiotics was initiated, pending blood culture results. Subsequently, the blood cultures returned positive for *Staphylococcus aureus*. Emergency coronary angiography was performed, which revealed thrombotic occlusion at the LAD. Thromboaspiration was performed, and, subsequently, drug-eluting stents (DESs) were implanted, with partial reperfusion results. TOE showed a 15 mm perivalvular abscess and an 8 mm vegetation in the right aortic cusp. CT angiography revealed thrombotic occlusion of the left axillary artery due to septic embolization. The outcome was unfavorable, with rapid death due to septic shock [20].

Maqsood K. et al. published an article describing a case of a 40-year-old man, with a history of intravenous drug abuse (IVDA), presenting to the emergency room with suggestive symptoms of infective endocarditis of the mechanical aortic valve prosthesis. The aortic valve was initially replaced following a long history for IE, and, subsequently, the patient developed another episode of recurrent endocarditis of the prosthetic valve, 2 years after implantation. The latter infection was with *Rothia* and *Stomatococcus* species. The initial TTE revealed a 10 mm vegetation on the prosthetic aortic valve, highly suggestive of endocarditis. Empirical treatment was initiated following the infectious physician’s recommendations, with vancomycin, gentamicin, and cefepime. The blood cultures were initially negative. Multidisciplinary collaboration with cardiovascular surgery considered the operative risk to be very high at the time, taking into account the patient’s comorbidities (previous stroke, recurrent IE) and IVDA. The second set of blood cultures grew *Candida albicans*. The patient received antifungal treatment for 27 days. TOE showed a suggestive mobile vegetation formation, 1.12 cm long, attached to the ventricular aspect of the aortic valve. Subsequently, the patient developed anterior STEMI, which raised the suspicion of an LAD occlusion. The lesion was confirmed by coronary angiography, which revealed occlusion at the mid-LAD. Thromboaspiration and a subsequent balloon angioplasty were performed. The post-procedural course was complicated with a symptomatic embolic stroke and aphasia 24 h after the thromboaspiration procedure. The patient was discharged to a palliative care hospital for the completion of 6 weeks of high-dose intravenous micafungin, with plans to follow a lifelong suppression therapy with fluconazole. However, the outcome was unfavorable, and he died 2 weeks later [21].

Inayat F. et al. published a case of a 69-year-old male who presented with intermittent fever and increased urinary frequency, having a prior history of *Streptococcus sanguinis* tricuspid valve endocarditis, aortic valve replacement, coronary artery disease and coronary aortic bypass grafting, atrial fibrillation, and diabetes mellitus. TOE revealed an image of a vegetation attached to the prosthetic aortic valve and a perivalvular abscess. The initial antibiotic management included levofloxacin, before switching to vancomycin and gentamicin for suspected endocarditis. The patient subsequently developed acute pulmonary edema and hypoxia, with a subsequent diagnosis of NSTEMI. A coronary angiography revealed 99% occlusion in the left main coronary artery (LMCA) and in the proximal left coronary circumflex artery (LCX), as well as a chronic total occlusion of the LAD and the right coronary artery (RCA), with a patent left internal mammary artery graft to the LAD artery and a patent saphenous vein graft to the posterior descending artery being noted. The blood cultures grew *Enterococcus faecalis*, confirmed also by the emboli culture, leading to treatment with ampicillin and gentamicin. The patient underwent aortic valve revision, abscess drainage, and left main coronary artery embolectomy. After being discharged in stable conditions, he completed 6 weeks of intravenous ampicillin therapy at a skilled nursing home facility [22].

  2. IE on the Native Aortic Valve

Khiatah B. et al. published a case, in 2020, of a young man, only 22 years old, with IE of the congenital bicuspid aortic valve. This had been the only case in the literature, up until that point, of STEMI complicated with ventricular fibrillation at the onset. Emergency coronary angiography revealed the complete occlusion of the proximal RCA, for which an angioplasty with bare metal stent implantation was performed, with optimal final results. The patient had been treated with antibiotics for alleged pneumonia the previous month. Other risk factors for acute coronary syndrome were not found in this patient. The echocardiography revealed a bicuspid aorta valve, with an attached, mobile formation of 0.9 × 1.7 mm, severe aortic regurgitation, and severe mitral regurgitation. The blood cultures were positive for *Abiotrophia defectiva*. The patient received empirical antibiotic therapy, pending the result of the blood cultures, and then targeted antibiotic therapy. He also underwent aortic and mitral valve replacement, with good final results [23].

Hibbert B. et al. published a similar case of IE on the bicuspid aortic valve, complicated by STEMI and ventricular fibrillation, in a patient who initially experienced septic shock. Coronary angiography showed LAD occlusion. An echocardiography noted the bicuspid aortic valve and the abscess of the artic root. The blood cultures grew the *Staphylococcus* species [24].

Ghazzal A. et al. also described a rare case of STEMI in a 38-year-old male with IVDA, who was diagnosed with aortic valve endocarditis with *Candida* species. An echocardiography objectified a vegetation of 9 × 9 mm attached to the aortic valve and an abscess of the aortic valve root extending to the interventricular septum. A surgical intervention was performed, with extensive debridement of the ring abscess with bovine pericardium patch repair, and they replaced the aortic valve with a biological valve of 23 mm. The postoperative course was complicated by a complete atrioventricular block that required the implantation of a permanent pacemaker. He was later transferred to a treatment center for the remainder of the 6-week micafungin therapy, followed by lifelong oral fluconazole suppression [25].

Bolton A. et al. published a case of IE of the native aortic valve complicated with multiple instances of embolization in the coronary and cerebral arterial territory. *Methicillin-susceptible Staphylococcus aureus* (MSSA) was isolated in the blood cultures. During the echocardiography, there was evidence of vegetation of the aortic valve that intermittently prolapsed at the level of the RCA ostium and was complicated with STEMI. In addition, the patient was diagnosed with a hemorrhagic stroke. A surgical intervention was performed with aortic valve debridement, without prior coronary angiography, with a favorable postoperative course [26].

Dina A. J. et al. wrote an article about a 42-year-old patient with a history of intravenous methamphetamine and heroin use, who was diagnosed with IE of the aortic valve and NSTEMI. During the physical examination, the patient was hypotensive and tachycardic, and Janeway’s lesions were observed on their fingertips. An echocardiography revealed vegetation on the aortic valve, with mild aortic regurgitation. The patient received a broad-spectrum empirical antibiotic treatment with vancomycin and cefepime. The heart team was still considering the best therapeutic decision for the patient, when he suddenly developed cardiorespiratory arrest and died despite all resuscitative efforts. Later, the blood cultures came back positive for *S. hominis* [27].

- Infective Mitral Valve Endocarditis Complicated with Septic Coronary Artery Embolization and AMI

Ściborski K. et al. describe the case of a 40-year-old man with no cardiovascular risk factors who presented to the emergency department with STEMI and was subsequently diagnosed with IE of the mitral valve with *Streptococcus salivarius*, for which he received an antibiotic treatment. Septic embolization occurred in two arterial territories in this case: both in the coronary arteries and in the spleen. The emergency primary percutaneous coronary intervention on the LAD was performed with the implantation of two drug-eluting stents (DESs). He subsequently underwent surgery for mitral valve replacement, due to the severe mitral regurgitation caused by the vegetation at this level, and a splenectomy for his splenic infarction. According to current recommendations from the AHA and ESC, splenectomy is recommended before cardiac valve replacement, as newly implanted prostheses carry a high risk of infection if all infectious foci are not eradicated beforehand [13,50]. In this case, an echocardiography was performed 12 months postoperatively, and an “additional chamber” was detected during the echocardiography, without communication with the other cardiac chambers and with a reduced ejection fraction of the left ventricle. The patient had no angina, showed no clinical or laboratory signs of inflammation, and did not present with symptoms of heart failure. However, a surprise came with the coronary angiography, which revealed two giant coronary artery aneurysms (46 × 32 mm and 23 × 20 mm) at the site of the previous stent implantation, with a slow blood flow in the LAD. Subsequently, computed tomography was performed to better analyze their morphology, and an interventional treatment was opted for due to the risk of surgical complications, with favorable evolution after 3 months and a decrease in aneurysm size during the follow-up computed tomography [32].

Another case of STEMI due to embolization from IE of the mitral valve complicated by a mycotic aneurysm was published in 2023 by Toda K. et al., featuring a 33-year-old woman who presented with cardiac arrest. The coronary angiography revealed the total occlusion of the LMCA. A balloon angioplasty followed by stent implantation at this level was performed, with a good outcome. The echocardiography showed severe mitral regurgitation and vegetation suggestive of endocarditis attached to the mitral valve. The blood cultures were positive for *Abiotrophia defectiva*. The patient underwent surgical intervention for mitral valve replacement. At the one-month follow-up, a coronary angiography detected a giant aneurysm of the coronary artery at the site of stenting, despite an appropriate antibiotic treatment for IE with *Abiotrophia defectiva*. The aneurysm measured approximately 5 mm in diameter and was located on the myocardial side of the LMCA bifurcation. Initially, a conservative treatment was chosen, but two weeks later, a coronary CT showed rapid growth of the aneurysm, raising concerns about the high risk of fatal rupture. A multidisciplinary team decided on a surgical coronary bypass and the ligation of the anterior and posterior ends of the aneurysm, with a subsequent favorable evolution [33].

On the other hand, Banerjee S. et al. published a case in 2023 of a young African American woman, 27 years old, diagnosed with Libman–Sacks endocarditis complicated by NSTEMI. The patient’s history included systemic lupus erythematosus (SLE). She presented to the hospital with typical anginal pain, but she also described blurred vision for approximately 6 months and swelling in both legs. A pulmonary embolism or aortic dissection was ruled out in the emergency department via computed tomography. The TTE revealed severe mitral regurgitation with a posterior jet, and the laboratory tests showed elevated cardiac enzymes, with a normal electrocardiogram. A coronary angiography demonstrated an occlusion of a branch of the posterolateral artery. Subsequently, TOE was performed, revealing sessile densities on the leaflet tips of the mitral valve at the coaptation points, confirming severe mitral regurgitation and no evidence of an atrial septal defect. The blood cultures were negative. The patient underwent mitral valve replacement with a good outcome. The case was interpreted as consistent with SLE endocarditis. A renal biopsy was performed, revealing lupus membranous nephritis. In the multidisciplinary team, the rheumatology consultant recommended treatment with corticosteroids. An ophthalmologic consultation was requested for visual changes, recommending a brain MRI, which revealed cystic encephalomalacia and surrounding gliosis along the watershed anterior cerebral artery/the middle cerebral artery territories symmetrically distributed in both hemispheres of the brain, related to the sequelae of remote ischemic changes. Additionally, no suggestive changes in acute ischemic events, possibly of embolic etiology, were observed. The neurological consultation concluded that the patient had an acute ischemic stroke due to embolization from Libman–Sacks endocarditis. During hospitalization, the patient’s condition improved with anticoagulant, antiplatelet, beta-blocker, corticosteroid, and statin therapy. Subsequent monitoring was conducted in the outpatient setting [34].

Carvalho Gouveia C. et al. published a case of a 69-year-old woman with multiple cardiovascular risk factors, including diabetes, who presented at the hospital for fever, asthenia, and myalgia. She was diagnosed with mitral valve endocarditis with multiple instances of embolization to the coronary arteries, the spleen, and the brain. Being diabetic, the patient did not show symptoms of acute coronary ischemia, but her myocardial necrosis enzymes were intensely positive, and the electrocardiogram was suggestive of STEMI. She underwent coronary angiography, which revealed a subocclusion at the level of the middle segment of the LAD. The brain CT showed weak cortico-subcortical hypodensities in the right and left frontal parietal areas. A thoraco-abdomino-pelvine CT was also performed to investigate a possible arterial embolism, and it described splenic infarcts. Subsequently, TOE was performed, which revealed severe mitral regurgitation with mobile vegetation, attached to the mitral valve, 18 × 10 mm. The blood cultures remained negative. The patient underwent mitral valve replacement surgery with mechanical valve and coronary artery bypass grafting, with an internal mammary artery to the LAD. The culture and biopsy of the vegetation were also negative. The postoperative evolution was complicated by cardiogenic shock, which required treatment with dobutamine and norepinephrine for five days. After obtaining a more detailed history, the patient reported that she was stung by a sea urchin before the onset of the disease, concluding that the most likely etiological agents were either the *Staphylococcus* spp. or the *Streptococcus* spp. The subsequent evolution was favorable under a treatment with ceftriaxone and ampicillin. Subsequently, after four weeks of combined treatment with ampicillin and ceftriaxone, the patient was discharged, with the recommendation to continue linezolid treatment for another two weeks to complete six weeks of antimicrobial therapy [35].

- Infective Tricuspid Valve Endocarditis Complicated with Septic Coronary Artery Embolization and AMI

Alla R. et al. have described a case of infectious endocarditis of a tricuspid valve prosthesis and the mitral valve, complicated by STEMI, in a 34-year-old woman, with a history of IVDA and a valvular prosthesis for recurrent endocarditis. Upon presentation to the hospital, the patient was in septic shock, requiring treatment with antibiotics and vasopressors. Multimodal imaging revealed embolization in multiple territories with splenic infarction, affecting the left kidney, and an ischemic area in the parietal and temporal lobes with concomitant subarachnoid hemorrhage. The TTE revealed an image suggestive of vegetation on the mitral valve, as well as a new stenosis of the recently replaced tricuspid valve. The blood cultures identified *Methicillin-resistant Staphylococcus aureus* (MRSA) and *Streptococcus agalactiae*. Although the patient had a significant increase in troponin along with ST elevation in leads V3–6 in the ECG, the cardiological consultation who had been requested for the diagnosis of STEMI did not recommend the intervention of coronary angiography due to the presence of multiorgan insufficiency, subarachnoid hemorrhage, and thrombocytopenia. The case was managed conservatively [46].

Cohen S. et al. published an article describing the case of a 26-year-old woman with IVDA, who presented with a picture suggestive of infective endocarditis and the course complicated by a STEMI. The diagnosis was confirmed by the TTE, which revealed a suggestive image of vegetation, large size, 2.4 × 1.4 cm at the tricuspid valve level. The physical examination revealed classic physical skin findings of IE: Osler’s nodules on her hands and Janeway’s lesions on her toes and on the soles of the feet. A coronary angiography revealed distal LAD occlusion and the occlusion of a small distal segment of the right posterior descending artery without atherosclerotic changes. Thromboaspiration followed by balloon angioplasty was performed. A brain CT was performed, which showed several areas of hyperdensity in the right frontal lobe and the left frontoparietal junction, which were consistent with subarachnoid hemorrhages, most likely resulting from septic embolism. The patient was electively intubated and transferred to a higher level of care facility for surgical management, where she continued antibiotic treatment. The initial blood cultures were positive for MSSA. Subsequently, the patient required continuous renal replacement therapy and a tracheostomy [47].

- Infective Endocarditis of Mitral–Aortic Junction Complicated with Septic Embolization of Coronary Arteries and AMI

Cardoso Monti Sousa L. L. et al. published a special case of a 19-year-old man known to have had a subvalvular aortic stenosis surgically corrected with a mechanic aortic valve prosthesis, who presented with a suggestive picture of infective endocarditis with STEMI symptoms. The intravenous administration of empiric oxacillin and ceftriaxone was started, pending the result of the blood cultures, which eventually identified *Aggregatibacter aphrophilus*. Subsequently, intravenous antibiotic therapy with ceftriaxone alone was continued for 6 weeks. TOE at admission showed severe mitral regurgitation and a round area with a reduced echo density at the mitral–aortic junction, suggestive of an abscess, without aortic valve prosthetic dysfunction. For STEMI, a coronary angiography was performed, which showed the complete occlusion of LAD without signs of atherosclerosis. A thrombectomy with aspiration, without stent implantation, was performed, with good results and a favorable evolution. Later, the patient was subjected to surgical intervention with the reconstruction of the mitral–aortic junction with a bovine patch and the reimplantation of a mechanical aortic valve [48].

## 3. Discussion

In our review of case presentations, we noted variations in the treatment methodologies, limitations in access to the state-of-the-art treatment for the presented cases, a variable and individualized approach to the management of each case, depending on the associated comorbidities, the availability or the lack of interdisciplinary collaboration, variable timings for transfer, and variable access to higher-level care centers when the cases decompensated clinically. In addition, there was heterogenicity in the adoption and use of the latest therapeutic methods, perhaps due to the rapid evolution of the diagnostic and treatment methods and their availability at the time of each case report.

### 3.1. Current Role and Challenges

#### 3.1.1. The Etiopathogenesis of Acute Myocardial Infarction Induced by Endocarditis through Septic Embolization

AMI type 1 is the most common type of AMI, according to the latest ESC Guidelines published in 2018, addressing the fourth universal definition of AMI [51]. It occurs by rupture of the atherosclerotic plaque with corresponding thrombosis and occlusion of the blood vessel. However, although rare, other causes of AMI can also be mentioned and should be considered as a differential diagnosis, especially when coronary angiography does not detect atherosclerotic plaques. One of these rare causes is the embolization of vegetation associated with IE. The incidence of infectious endocarditis is 3–10 per 100,000 people. The clinical presentation of IE is nonspecific and varied, with the most common findings being the presence of a heart murmur and fever. Other symptoms may include weight loss, sweating, malaise, fatigue, splenomegaly, and skin manifestations (hemorrhages and petechiae), but also signs of complications such as sepsis, heart failure, and, last but not least, systemic embolization. In these cases, the mortality rate at discharge is over 40%, and the prognosis is poor [28]. ACS that complicates infectious endocarditis is a rare complication, with an incidence of 2.9% and a mortality rate which can reach up to 64% [29,31]. Usually, coronary embolism occurs in the first 15 days of the disease [35]. Our literature review indicates that, in patients with large vegetations and prosthetic valves, embolic phenomena are often recurrent. There is no standard management strategy for these cases, as their incidence is limited. The current guidelines offer firm recommendations on the empirical antibiotic treatment for valvular endocarditis. Subsequently, the treatment should be adjusted according to each pathogen, the severity of the infection, the models of antibiotic resistance, and the type of valve (native or prosthesis). It must also include treatment with bactericidal antibiotics for a long period [35]. Surgical embolectomy or emergency percutaneous thrombectomy, balloon, or stent angioplasty is recommended if thrombectomy fails [21,28,47]. Thrombolytic agents have often been used in ACS from septic emboli, but an increased risk of intracerebral hemorrhage due to associated mycotic cerebral infarcts has been observed [21,22,47]. Another rare cause of AMI without atherosclerotic plaque is extrinsic compression. Specialty studies mention that another cause of ACS may be secondary to coronary artery compression by perianular complications, including the development of aggravated abscesses and pseudoaneurysms [19]. A case of NSTEMI through extrinsic compression by a pseudoaneurysm of the aortic endocardium is described by Acar Z. et al. [52]. Additionally, in patients younger than 35 years old, the cause of STEMI without atherosclerotic disease is most likely related to hypercoagulability, such as factor V Leiden, antiphospholipid syndrome, or nephrotic syndrome. The traditional risk factors for atherosclerotic coronary artery disease (CAD) are dyslipidemia, hypertension, smoking, diabetes mellitus, and family history of CAD. In young patients, the non-atherosclerotic causes of STEMI may be vasculitis, spontaneous coronary artery dissections, or septic emboli from an infected heart valve. When a doctor is faced with a young patient with STEMI, they should actively seek all these factors to avoid delaying the diagnosis and treatment [23].

Specialized studies support the etiology of coronary artery mycotic aneurysm (CAMA) attributed primarily to IE or anterior interventions in the coronary arteries, particularly in patients without immunosuppressive conditions. In addition, studies suggest that there have been approximately 100 reported cases of CAMA to date, with over 80% of these being associated with coronary stent implantation. While coronary aneurysms can be asymptomatic, some patients, especially those with giant CAMA, may experience hemodynamic changes due to a slowed and turbulent blood flow. The outcomes can be devastating, ranging from simple exertional angina to ACS. Another pathogenesis of ACS in the case of giant aneurysms is thrombosis within their lumen, which can lead to distal embolization and myocardial infarction or even the compression of adjacent structures. Another serious complication of CAMA can be cardiac tamponade, due to the rupture of the aneurysm. As there is currently no consensus, the treatment options for giant CAMA may include a conservative approach or cardiac surgery with the ligation of the aneurysm, resection, a graft bypassing of it, and percutaneous interventions (covered stent, coil, stent-assisted coiling). The choice of the correct treatment depends on the patient’s condition and the anatomy of the aneurysm [32,33,53].

#### 3.1.2. The Valve with the Highest Embolic Risk Leading to Acute Myocardial Infarction

There are conflicting data in the literature according to which a valve affected by IE is most commonly responsible for the development of septic embolic-induced AMI: there are studies that claim that the aortic valve is most commonly responsible for septic embolization in coronary arteries, with the subsequent development of AMI [19,21,27], similar to our findings. There are also previous studies suggesting that vegetations on the mitral valve have the highest embolic risk [25,35,48,54]. Other studies suggest that the anterior mitral valve has the highest embolic risk, at a rate of 37%, compared to general mitral valve vegetation, at 25%, followed by aortic vegetation, at 10% [22,25,55].

#### 3.1.3. The Coronary Artery Most Frequently Affected by Septic Embolization

Studies support that the most frequently affected coronary artery is the LAD (left anterior descending) [21,25,27,35,48,54]. A few cases that presented with sudden cardiac death have been described in the literature as being secondary to LMCA occlusion from septic embolisms in IE. The LMCA embolism is extremely rare and has been described as being associated with cases of native aortic valve or mitral valve endocarditis. Coronary embolisms secondary to endocarditis with Enterococcus most commonly originating from the aortic valve have been previously reported, and they have been associated with LCX occlusion and papillary muscle rupture [22]. Some cases described infective endocarditis with rare embolization of the posterior descending artery [29,47]. Embolization in multiple arterial territories is also described in the literature, such as embolization from the aortic valve to the coronary arteries, concomitant with embolization to the arteries of the upper limbs [20] and embolization from the mitral valve to the cerebral, splenic, and spinal levels [4].

#### 3.1.4. Pathogens Involved in Septic Embolization from Endocarditis

A literature review points out that, in the United States, the most common pathogen involved in septic embolization is *Staphylococcus aureus* (31%); however, other organisms may be involved, with various frequencies, including *Streptococcus viridans* (17%), *Staphylococci coagulase-negative* (CoNS) (11%), *Enterococci* (11%), *Streptococcus gallolyticus* (formerly *Streptococcus bovis*) (7%), other *Streptococci* (5%), Haemophilus, Aggregatibacter, Cardiobacterium, Eikenella, Kingella (HACEK) organisms (2%), Aggregatibacter, Cardiobacterium, non-HACEK Gram-negative bacteria (2%), and fungi (2%). Rarely, *Staphylococcus hominis* can cause IE, as it is a coagulase-negative staphylococcus which is part of the normal skin flora [27]. Other studies name the most common etiological agent *Staphylococcus aureus* (26.6–40%), followed by the viridian group streptococci and *Enterococcus* spp. [35]. *Abiotrophia defectiva* is a streptococcus that cannot grow on common culture media. This is a virulent bacterium, with demanding in vitro growth requirements, which particularly affects endovascular structures and is also involved in numerous cases of endocarditis with negative common blood cultures. *Abiotrophia defectiva* has an excessive embolization rate from valvular vegetation compared to other microorganisms and quickly leads to massive valvular destruction and heart failure [56]. Fungal endocarditis has a low incidence of <2% of all cases of infectious endocarditis. Another less common microorganism in IE is Candida. *Candida albicans* is the pathogen involved in about 24% of cases of fungal endocarditis. Specialized studies have confirmed that fungal endocarditis is more common in patients with prosthetic valves, indwelling venous catheters, post heart surgery, or in cases of immunocompromised patients, such as in the case of patients with IVDA. The guidelines recommend antimicrobial therapy, including intravenous antifungal medications, followed by the long-term use of a suppressive oral antifungal agent because of an increased risk of recurrence [25,47].

#### 3.1.5. Embolic Events from Endocarditis on the Right-Side Valves

IE on the right side is more commonly associated with IVDA, intravenous catheters, and intracardiac devices [20,35].

The incidence of infectious endocarditis associated with IVDA has increased significantly over the past decade, according to a state-of-the-art review published in the *Journal of the American College of Cardiology* (*JACC*) [57].

#### 3.1.6. Septic Embolic Events from Endocarditis on the Left-Side Valves or Prosthetic Valves

Scientific articles suggest that a significant proportion of IE is associated with the presence of a prosthetic valve. This accounts for 10–30% of the total IE cases and involves both mechanical and biological valves. In particular, for biological valves, IE may occur in the first 3 months after prosthetic surgery. IE has a predisposition for left heart valves, with an incidence of 90–95% of all cases.

When blood cultures are negative in patients with severe valvular regurgitation, apparently without a detectable cause, and the patient has an autoimmune disease, suspicion should be raised for Libman–Sacks endocarditis (LSE). The specialized literature describes this entity as a non-infectious endocarditis that affects 1 in 10 patients with SLE. Most often, the verrucous vegetation affects the mitral and aortic valves. The incidence of cerebrovascular events due to embolism from LSE has been reported in studies to be between 10% and 20% [34].

### 3.2. Multidisciplinary Collaboration in Infective Endocarditis Complicated by Septic Embolic-Induced Acute Myocardial Infarction

**Figure 6:**
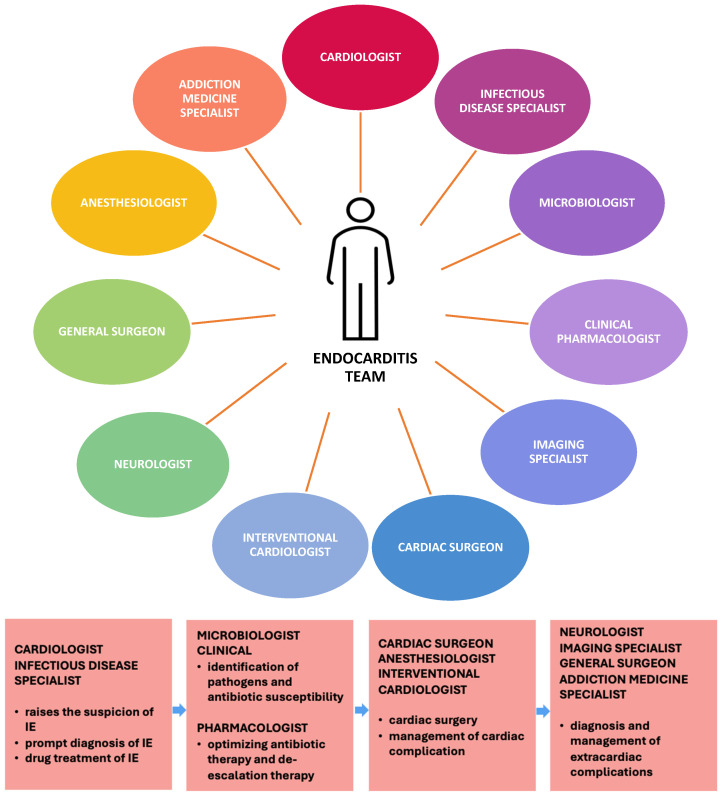
The role of the multidisciplinary team in managing endocarditis.

Many aspects of the diagnosis and treatment of infective endocarditis involve complex steps, such as utilizing multiple imaging techniques, selecting the most suitable antimicrobial therapy, managing both cardiac and extra-cardiac complications, and determining when cardiac surgery is necessary. When endocarditis is complicated by acute myocardial infarction, the situation becomes even more sensitive, requiring the involvement of interventional cardiologists. To efficiently address these challenges, collaboration among specialists from different fields is crucial, including cardiologists, infectious disease experts, cardiac surgeons, microbiologists, and imaging specialists (cardiac, nuclear, and neurological) (Figure 6). This multidisciplinary approach, known as the endocarditis team, has proven essential for optimizing patient care and reducing mortality rates. It is recommended that each tertiary center should establish an endocarditis team separate from the standard heart valve team, as described in the 2015 guidelines of the European Society of Cardiology [12]. Therefore, we consider important to emphasize the significance of the endocarditis team as an example of multidisciplinary collaboration, as it is addressed in this review.

### 3.3. Preoperative Complications Associated with IE Complicated by Septic Embolic-Induced AMI

According to the latest ESC guidelines on infective endocarditis from 2023, infective endocarditis is associated with certain risks and complications [13]. But, when IE is associated with a rare complication—acute myocardial infarction—the mortality rate at discharge increases greatly [10].

#### 3.3.1. Preoperative Cardiac Complications: Heart Failure, Cardiogenic Shock, and Conduction Abnormalities

1. In IE, the pathophysiology of heart failure (HF) can be attributed to several mechanisms and is multifactorial. When discussing IE complicated by AMI through septic embolization, all these mechanisms are aggravated. Valvular dysfunction in IE is worse when IE is complicated by AMI through septic embolization, via two mechanisms which cause valve regurgitation volume overload due to IE, but also valve regurgitation due to AMI. In septic embolization from IE, the incidence of heart failure is increased to 70%, and mortality is doubled [58]. One of the most frequent causes of HF include leaflet perforation and rupture, along with mitral chordal rupture, resulting in the development of severe valvular regurgitation or the exacerbation of pre-existing valvular regurgitation [12,13]. Other less frequent causes of HF include the obstruction of the leaflet opening and closure by the vegetation mass, intracardiac fistulas, or myocardial infarction caused by vegetation embolizing into the coronary arteries [13,14].

The diagnosis of HF complicated by septic embolic-induced AMI is primarily based on clinical presentation and ECG, confirmed with an echocardiography. Other diagnostic tools, such as coronary angiography and cardiac biomarkers (troponin and B-type natriuretic peptide), may also be used in the perioperative period to assess circulatory dysfunction and the extent of myocardial damage [13,58].

The treatment of HF complicated by septic embolic-induced AMI requires a multidisciplinary approach and may include inotropic support and mechanical ventilation. Antimicrobial therapy is also essential for treating the underlying infection. The decision regarding the timing of surgical intervention in this case should be made by the endocarditis team [2,14,58].

Regarding the management of AMI induced by septic embolization of the IE, there are no firm recommendations in the literature due to the rare occurrence of septic coronary embolisms in patients with IE. There are only treatment suggestions in the literature, including manual thrombus aspiration [59], percutaneous revascularization with thrombectomy, balloon dilatation with or without the placement of a coronary stent, surgical embolectomy [15,16], or (emergency) aortic valve replacement if the coronary compression is caused by an aortic abscess. There are studies suggesting that fibrinolytic therapy can lead to severe intracranial hemorrhage in patients with IE complicated with AMI through septic embolization and is, therefore, contraindicated [59].

2. Cardiogenic Shock

Cardiogenic shock (CS), the most severe form of HF, can occur when compensatory mechanisms fail in acute HF. The in-hospital mortality due to CS in IE exceeds 50% and is strongly associated with adverse outcomes. Clinical presentation underlies the diagnosis of CS, subsequently confirmed by invasive hemodynamic monitoring [45,58].

Echocardiography plays an extremely important role in the diagnosis and assessment of cardiac dysfunction and valvular dysfunction.

The treatment of CS in IE complicated with AMI by septic embolization includes inotropic support and mechanical ventilation. Treatment requires aggressive interventions and prompt recognition. The decision of an emergency or salvage surgery is made by the endocarditis team, aiming to address the underlying valve dysfunction but also the coronary artery responsible for the AMI [13,14,45,58].

3. Conduction Abnormalities

The occurrence of conduction abnormalities, such as arrhythmias or heart blocks, in IE complicated by septic embolic-induced AMI may have a dual mechanism: it may be the consequence of the obstruction of the vessel irrigating the atrioventricular node or the extension of the infection beyond the valve annulus. The atrioventricular block may occur not only as a complication of the paravalvular extension of the infection but can also develop as a consequence of AMI [2,58].

The diagnosis of conduction abnormalities in IE complicated with AMI by septic embolization is based on clinical presentation, and, subsequently, ECG confirms the diagnosis.

Treatment includes temporary pacing or the implantation of a permanent pacemaker, as well as a temporary pharmacological treatment. Emergency surgical indication is maintained in the case of severe conduction disorders such as atrioventricular block, as this presents an increased risk of adverse outcomes [12,13,58].

#### 3.3.2. Preoperative Non-Cardiac Complications: Uncontrolled Infection/Sepsis/Septic Shock and Neurological Complications, including CEREBRAL Embolization, Splenic Embolization, Pulmonary Complication, Mycotic Aneurysm, Osteoarticular Infections, and Acute Renal Failure

1. Septic shock represents a particularly deadly complication of IE, affecting around 5–10% of patients. The risk factors for septic shock include infections caused by Gram-negative bacteria and *S. aureus*, persistent bacteremia, acute renal failure, the acquisition of infection in healthcare settings, the presence of large vegetations, emboli in the central nervous system, and diabetes mellitus [13].

The diagnosis is made in the presence of the need for vasopressor support to maintain a mean arterial pressure above 65 mmHg and with the assistance of biomarkers such as serum lactate, which should be >2 mmol/L [12,13,14].

The treatment of critically ill patients requires complex decisions and includes supportive therapy for organ dysfunction, the appropriate antimicrobial, and, eventually, surgical interventions. In cases where contraindications and indications for cardiac surgery may coexist, multidisciplinary collaboration is crucial [58].

2. The risk of embolism in IE is substantial, affecting almost 50% of patients with IE. The brain is the most common site of extra-cardiac embolism for left-sided IE [13]. Ischemic vascular complications are the main neurological complication in IE, accounting for 70%, with intracerebral hemorrhage accounting for 10% and intracerebral abscess, meningoencephalitis, and subarachnoid hemorrhage each accounting for an equal percentage of 5% [60].

To prevent neurological complications, prompt diagnosis of IE and the initiation of antibiotic therapy are necessary. Early cardiac surgery in high-risk patients is essential to prevent complications related to septic embolization.

When neurological complications are suspected, cerebral imaging is mandatory. The evaluation should include an MRI with and without gadolinium or a CT with and without contrast if the MRI is not feasible. The MRI often detects “silent” lesions in patients without neurological symptoms [7,8,58,59,60].

3. Embolization can affect any organ, but the most common organ involved, besides the brain, is the spleen, followed by the kidneys, the extremities, and the skin [7,58].

The spleen-related complications associated with IE range from asymptomatic infarction and abscess formation to splenic rupture and cardiovascular collapse. Splenic infarctions are common and often asymptomatic. Up to 5% of splenic infarctions can progress to abscess formation [13].

One diagnostic tool for these complications is CT imaging, including whole-body CT (i.e., chest, abdomen, and pelvis) and cerebral CT [13,58].

For treatment, coronary angiography, splenectomy, surgical embolectomy, percutaneous abscess drainage, or endovascular interventions may be required in some cases, with a concurrent supportive and antimicrobial treatment [2,13,14,58].

4. Pulmonary embolism for right-sided IE and cardiac pacemaker leads is frequent, but there are extremely few data on concomitant pulmonary and coronary embolization. In IE, the incidence of pulmonary complications is between 4 and 10% [16,58].

The diagnostic methods in IE consist of chest CT for pulmonary embolisms.

There is no consensus supporting a certain treatment for the pulmonary complications in IE associated with AMI: pharmacological treatment also includes antimicrobial therapy and supportive care, and, in severe cases, endocarditis team referral is required.

5. An infective (mycotic) aneurysm is a rare-yet-potentially severe complication of IE. The treatment options for infective cerebral aneurysms include antibiotic therapy alone or in combination with endovascular or surgical interventions.
6. Osteoarticular infections are rarely associated with IE. Myalgia and back pain are reported in 12–15% of cases [13]. Arthralgia occurs in approximately 10% of patients, sometimes affecting multiple joints sequentially. Rheumatologic conditions and musculoskeletal symptoms resolve rapidly and completely with antibiotics, and their presence does not impact the prognosis of IE [12,13].
7. With respect to the acute renal failure associated with IE, several factors may contribute to the worsening or onset of renal dysfunction: (i) renal infarction due to septic embolism; (ii) immune complex and vasculitic glomerulonephritis; (iii) nephrotoxicity of the contrast agents used for diagnostic imaging; (iv) hemodynamic insufficiency in patients with heart failure; and (v) antibiotics’ and other medications’ toxicity (particularly associated with aminoglycosides, oxacillin, vancomycin, amoxicillin, and the concurrent use of diuretics and/or nonsteroidal anti-inflammatory drugs). Severe renal failure requiring hemodialysis has been reported in 6% of patients with IE and is associated with a very high risk of mortality (40%) [13,14].

### 3.4. Perioperative Complications Associated with IE Complicated by Septic Embolic-Induced AMI

#### 3.4.1. Intraoperative Complications

1. Bleeding and Coagulopathy

Hypercoagulability is present in IE in general. During cardiac surgery for IE, however, the occurrence of severe bleeding due to severe coagulopathy is common. The exact incidence of coagulopathy and bleeding during cardiac surgery for IE complicated with AMI is not known.

Studies report that up to 80% of patients undergoing valve replacement surgery receive the transfusion of blood products.

The management of bleeding and coagulopathy in IE includes the transfusion of blood products, including fresh frozen plasma, packed red blood cells, and platelets, according to guidelines [58].

2. Hemodynamic Instability

Hemodynamic instability during surgery for IE complicated with AMI via septic embolization may have various causes, including systemic inflammatory response and the impaired cardiac function of the patient.

The diagnostic methods of hemodynamic instability include (extensive) hemodynamic monitoring, including cardiac output measurement and invasive blood pressure monitoring. In addition, intraoperative transesophageal echocardiography plays a crucial role in the assessment of valvular or ventricular dysfunction. Also, the monitoring of oxygen saturation, serum lactate, and urine output assesses peripheral organ ischemia.

The treatment of hemodynamically unstable situations may include inotropic and vasopressor support as well as aggressive fluid resuscitation. Since there is no valid consensus for the treatment of hemodynamic instability during the operation of infective endocarditis complicated with AMI, hemodynamic management should be based on general pathophysiological mechanisms [58].

#### 3.4.2. Postoperative Complications

1. Permanent Pacemaker Requirement

Heart surgery can cause changes in the conduction system. A diagnosis is made by continuous postoperative ECG monitoring. The treatment includes temporary pacing until the optimal time for permanent pacemaker implantation is chosen, which must be within an infection-free range [61].

2. Postoperative Stroke

Postoperative stroke can occur due to embolisms from calcified plaques or valve handling with attached vegetation. Postoperatively, the incidence of stroke is variable, between 2 and 11% [49,62]. The diagnosis of postoperative stroke is made by close clinical examination upon awakening. The diagnosis is confirmed and localized by MRI or CT imaging. Treatment is limited for postoperative strokes. Endovascular mechanical thrombectomy and/or intra-arterial thrombolysis may be performed after a careful evaluation of the benefits and risks [63].

3. Re-exploration Due to Bleeding

The causes of surgical re-exploration can be multifactorial: a consequence of coagulopathy, primary surgical origin, or a combination of both. The incidence of surgical re-exploration when it comes to infective endocarditis is reported to be between 8 and 12% [58].

Diagnosis hinges on the visualization of the increased amount of postoperative chest drainage. A diagnosis may also be clinical, through signs of cardiac tamponade. Echocardiography confirms the diagnosis with the visualization of pericardial fluid, but the laboratory parameters and the hemodynamic status of the patient also help in the diagnosis.

The treatment is blood transfusions according to guidelines. In selected cases, subxiphoid blood evacuation or repeat sternotomy may be useful, where the systematic intraoperative inspection of potential bleeding sites is crucial [64].

### 3.5. Concomitant Non-Cardiac Complications Associated with IE Complicated by Septic Embolic-Induced AMI

Multiple septic embolisms from infective endocarditis are a severe complication of IE. Septic embolization in the coronary arteries concomitant with septic embolization in the brain and spleen is very rare. The diagnosis of stroke and splenic infarct/abscess is based on clinical manifestations and confirmed by imaging methods (e.g., CT). The diagnosis of acute coronary syndrome is based on coronary angiography. As a treatment method, anticoagulants are recommended only if there is an indication, e.g., prosthetic valve, but not routinely recommended due to the risk of bleeding or vascular rupture. No studies and no consensus are clarifying this need for uniform treatment in these patients. Multiple embolisms caused by large vegetations on the LVOT are very difficult to control, so surgical treatment should not be delayed [65,66].

## 4. The Antimicrobial Treatment of the IE

The latest 2023 endocarditis guidelines by the European Society of Cardiology [13] recommend that the antibiotic used for the first-line treatment should be de-escalated after bacterial isolation, according to the antibiotic susceptibility. The aim of the therapy is microbial eradication. Hemocultures are mandatory before antibiotic initiation. Bactericidal regiments are preferred, including synergistic drugs, such as aminoglycosides associated with beta-lactams or glycopeptides. The duration of treatment varies from 2 to 6 weeks for NVE and should be over 6 weeks in PVE.

### 4.1. The Initial Antibiotic Regimen for Infective Endocarditis Depends on the Place (Community or Nosocomial), the Category of Valve (NVE, Early, or Late Postsurgical PVE), the Previous Antibiotic Treatment, Local Antibiotic Resistance Data, and Regional Epidemiological Characteristics

In patients with community-acquired native valve endocarditis (NVE) or late prosthetic valve endocarditis (PVE) (≥12 months post surgery), ampicillin in combination with ceftriaxone or (flu)cloxacillin and gentamicin should be considered. In patients with early PVE (<12 months post surgery) or nosocomial and non-nosocomial healthcare-associated IE, vancomycin or daptomycin combined with gentamicin and rifampin may be considered. The alternative first-line antibiotic on beta-lactam allergic patients with community-acquired NVE or late PVE (≥12 months post surgery) should be a combination of vancomycin and gentamicin.

### 4.2. Targeted Antimicrobial Therapy Considers Etiological-Specific Recommendations, Based on Efficiency Data from Clinical Trials and Cohort Studies

However, the decision of an antibiotic regimen is decided according to the susceptible standard dose, the susceptible increased exposure, or the resistant category related to the EUCAST clinical breakpoints of MIC. The global alarming antibiotic resistance is increasing the rate of treatment failure. Another challenge is bacterial antibiotic tolerance, consisting in selected slow-growing and dormant microbes which are not resistant but persist in vegetations and biofilms after treatment discontinuation. Higher doses and prolonged courses of antibiotics could be treatment options but are limited by patients’ toxicity and tolerability [13].

The safest and most frequently used antimicrobials for IE therapy are beta-lactams, but their standard dosing could be subtherapeutic or correspond to toxic concentrations. The beta-lactam PK/PD data of experimental and animal models could not be equivalent to humans and are not considered by the treatment guidelines for dose individualization in IE therapy. The beta-lactam PK/PD is influenced by the interaction between an antibiotic and the vegetation, and the serum antibiotic concentrations may not reflect the concentrations within the vegetation. The ideal concentrations and the duration of administration are incompletely known but should be clarified for optimizing the present therapies [67].

Endocarditis related to cardiac electronic devices mostly involves CoNS and *S. aureus*, but other etiologies could be the *Enterococcus* spp., *Beta-hemolytic streptococci*, *Oral streptococci*, the *Corynebacterium* spp., *Gram-negative rods*, or polymicrobial agents; Fungal infections are rare; device extraction is required, but reimplantation is a difficult decision; and the antibiotic treatment should consist of combined regimens, directing MRSA and gram-negative germs [13].

## 5. Monitoring of Antibiotic Treatment in Infective Endocarditis

The aim of antibiotic therapy in endocarditis is achieving the clearance of blood cultures and the “sterilization” of vegetations, with the best possible efficacy, in the shortest time, and with minimal side effects. Therapeutic drug monitoring (TDM) leads to personalized medicine and also allows for the optimization of antibiotic prescribing. As endocarditis is a serious systemic infection, the recommendation of TDM include antibiotics with a narrow therapeutic index, suspected drug interactions, difficult-to-treat pathogens, persistent infections, renal impairment, suspected drug toxicity, patients with an extreme weight (over 120 kg, less than 50 kg, body mass index greater than 30 kg/m^2^), elderly people over 80 years of age, and other conditions that may cause pharmacokinetic changes [68]. TDM has emerged as a method to balance these goals, especially when using antibiotics with a narrow therapeutic window and potentially nephrotoxic side effects, like vancomycin and aminoglycosides. Different pharmacokinetic and pharmacodynamic parameters involving the minimal inhibitory concentration (MIC), the area under the 24 h plasma concentration–time curve (AUC), the serum and plasma concentration, and the duration of time during which the serum concentration exceeds the MIC have been utilized to classify antibiotics based on their effect on organisms: time-dependent ones (beta-lactams) or concentration-dependent ones (aminoglycosides, fluroquinolones, daptomycin, and vancomycin).

The percentage duration of time during which the serum concentration exceeds the MIC is used to predict beta-lactam antibiotics’ efficacy, with values of 60–70% predicting a bactericidal effect. There are studies showing that the TDM for beta-lactam antibiotics in endocarditis [69,70] could identify patients “overdosed” on these antibiotics at the currently recommended doses and that dose reduction based on TDM would have no impact on prognosis and the frequency of side effects. The current guidelines acknowledge that more studies are needed before recommending TDM for beta-lactam antibiotics [50].

The AUC/MIC can predict efficacy for glycopeptides (vancomycin, teicoplanin, telavancin, oritavancin) and lipopeptides (daptomycin) [50]. Vancomycin is frequently used in endocarditis therapy, being recommended both for empiric coverage before a pathogen is isolated and for definitive therapy for susceptible bacteria [13]. An adequate vancomycin concentration is necessary to maintain an adequate efficacy, prevent the emergence of bacterial resistance, and avoid vancomycin-induced toxicity. In 2009, the first consensus for vancomycin monitoring was published, and it recommended one to use the AUC/MIC > 400 mg·h/L as a predictor of vancomycin activity [71]. As the AUC was difficult to calculate initially, requiring repeated blood level draws, a serum trough concentration from 15 to 20 mg/L was considered an adequate surrogate marker in patients with MIC < 1 mg/L and normal renal function. The emergence of Bayesian software TDM has made AUC calculations less cumbersome, with software generating an accurate AUC with reduced blood draws. With the AUC being better at predicting the development of vancomycin-induced kidney failure than the trough levels, current society guidelines [68] recommend doctors to maintain an AUC between 400 and 600 mg·h/L (when the MIC is 1 mg/L) to maximize treatment efficacy and minimize vancomycin nephrotoxicity. Bayesian-derived AUC calculation is suggested, and trough-only monitoring is no longer recommended for vancomycin-treated MRSA infections [68]. With vancomycin MIC > 1 mg/L, daptomycin has been associated with better outcomes in MRSA bacteremia than vancomycin; thus, daptomycin is recommended for MRSA and enterococcal IE cases, initially at a higher dose of 10 mg/kg, in order to prevent antibiotic resistance [13]. Although observational reports about daptomycin TDM are available, to date, no study has evaluated the benefits of daptomycin dose adjustments versus fixed-dose therapies [72].

With respect to prosthetic valve endocarditis and therapeutic drug monitoring (TDM), Gallerani hypothesized the effective role of sequential dalbavancin therapy, guided by TDM, in patients with prosthetic valve endocarditis and severe infections of their intracardiac devices, who had limited surgical indications and were very difficult to treat, and confirmed the need for prospective studies to analyze this entity [73].

## 6. Future Directions

The first aspect that holds significant interest for future endeavors is aimed at reducing the occurrence of infective endocarditis and subsequently mitigating the risk of embolization. One of these preventative measures involves combating IVDA. As mentioned earlier, right-sided endocarditis commonly arises as a consequence of IVDA, and yet IVDA can also be linked to left-sided endocarditis [21,25,27,46,47]. The literature highlights a concerning statistic: approximately 15,000 patients are admitted annually in the United States due to endocarditis secondary to opioid use disorder. Tragically, the mortality rates among these individuals are alarmingly high, necessitating prolonged hospital stays and accumulating over USD700 million in annual hospitalization costs for completing the requisite six-week course of intravenous antibiotic therapy. Interestingly, despite individuals with IVDA often presenting with iron deficiency and anemia, administering iron supplementation paradoxically heightens their susceptibility to bacterial infections and exacerbates the severity of various infectious conditions [74]. Despite the existence of effective opioid-agonist therapies like methadone and buprenorphine, their continuity or initiation during and after hospitalization remains inadequate for most patients. Moreover, a significant proportion of patients fail to complete their prescribed regimen of intravenous antibiotics, a critical component for averting endocarditis-associated mortality and morbidity [75].

The second area of interest for future explorations seeking to mitigate complications like infective endocarditis involves delving into autoimmune diseases. In a previous discussion involving Banerjee S., a compelling case emphasized the importance of remaining vigilant for Libman–Sacks endocarditis (LSE) when managing patients with autoimmune disorders such as systemic lupus erythematosus (SLE), especially in the presence of valvular dysfunction. LSE typically manifests without symptoms; yet, its potential for grave complications necessitates prompt diagnosis and intervention [34]. Autoimmune diseases emerge when the body’s defense mechanisms mistakenly target its tissues, leading to structural and functional anomalies across diverse organs. A complex interplay of genetic predisposition, environmental factors, and immune dysregulation, sometimes triggered by infections, underpins the onset of these conditions. There exist nearly a hundred recognized autoimmune disorders, with some of the most prevalent ones including SLE, multiple sclerosis (MS), rheumatoid arthritis (RA), Graves’ disease (GD), type 1 diabetes mellitus (T1D), and inflammatory bowel disease (IBD). Also, there are cases in the literature of the association between thyroid autoimmune disease and infective endocarditis. Therefore, the investigation of thyroid status in the management of endocarditis remains extremely important. This association can be explained by two mechanisms. The first mechanism is that autoantigenic TSH receptor antibodies in Graves’ disease induce mucopolysaccharide secretion by the activation of endothelial cells, leading to myxomatous changes in the heart valves through their thickening, with the likelihood of infective endocarditis in transient bacteremia, which would not normally produce infection. Conversely, the second mechanism suggests that infections themselves can trigger autoimmune thyroid disorders by releasing antigens that are sequestered in the inflammation and molecular mimicry of infected structures. Although this association is extremely rare, cases of the concomitant presentation of infective endocarditis with thyrotoxicosis are described. Additionally, individuals with autoimmune disorders such as Hashimoto’s thyroiditis may face an elevated risk of certain infections, including endocarditis, as the autoimmune condition can compromise the immune system’s functionality. Cases of infective endocarditis and thyroid abscess or suppurative thyroiditis have also been reported [76,77].

As we navigate towards the future, one of the most pivotal directions, particularly starting from the year 2024, revolves around patient-centric healthcare, which emerges as the third area of future interest. Within the contemporary medical landscape, a notable paradigm shift is underway towards patient-centered care, prioritizing not just clinical outcomes but also the quality of life (QoL) experienced by patients. This shift has prompted a growing emphasis on the integration of Patient-reported Outcome Measures (PROMs) and Patient-reported Experience Measures (PREMs) into healthcare practices. PROMs encapsulate the outcomes reported directly by patients, including aspects such as QoL, pain levels, and levels of anxiety, providing crucial insights into the subjective impact of medical interventions. On the other hand, PREMs capture the patient’s firsthand experiences within the healthcare system. PREMs are further categorized into Relational PREMs, which delve into the dynamics between patients and healthcare providers, encompassing aspects such as expectations, preferences, and the fulfillment of healthcare goals, and Functional PREMs, which assess the operational efficiency and effectiveness of the healthcare system from the patient’s perspective [69,70,71,72,73,74,75,76,77,78,79,80,81]. Numerous studies have corroborated the significance of these patient-centered concepts, illustrating how factors like quality of care, effective communication, and the post-treatment experience can influence patient satisfaction and the overall healthcare outcomes, particularly in the context of endocarditis treatment [82].

The fourth area of interest for future research is artificial intelligence (AI) in infective endocarditis. AI is emerging as a powerful tool in various aspects of IE management. Firstly, it is revolutionizing the prediction of infective endocarditis [83]. By harnessing large datasets and advanced algorithms, AI systems can identify subtle patterns and risk factors that may not be readily apparent to clinicians, thereby enhancing the accuracy of predictive models. Secondly, AI holds promise in risk stratification in IE [84]. By analyzing complex patient data, including clinical, imaging, and laboratory parameters, AI algorithms can help stratify patients based on their risk of developing complications or experiencing adverse outcomes, enabling more personalized and targeted management strategies. Additionally, AI is shedding new light on the pathogenesis of IE and its complications, such as sepsis [85]. Through the sophisticated analysis of molecular interactions, genetic factors, and environmental influences, AI-driven research is unraveling the intricate mechanisms underlying these diseases, offering novel insights that could inform the development of innovative therapeutic interventions and preventive measures. As AI continues to evolve, its integration into IE research promises to accelerate progress in understanding, diagnosing, and managing this complex condition.

## 7. Limitations

This study has a few limitations. They are related to the standardization of the management of septic embolization in coronary arteries from infective endocarditis, a lack of clear evidence to support a uniform approach, and studies being based only on case reports or very small batches of patients. First, the number of articles in this review is small, as it pertains to how rare this complication of IE is encountered, and this can lead to biased samples. Second, we cannot assert that it is a thorough review covering all the studies in the literature, since cases published in languages other than English may describe similar patients. Third, our study is a retrospective review. As such, it is inferior to prospective studies and carries a risk of bias in patient selection, and, subsequently, the generalization of our findings could be limited. Finally, the guidelines for endocarditis have been changing over the years. These may be future research directions for cardiology and infectious disease research and may even—why not—lead to the conception of a new field of infectious–cardiac studies.

## 8. Conclusions

Following our analysis of the literature, we observed that our review aligns well with the existing clinical information on the subject. Infective endocarditis predominantly affects left-side heart valves, with the aortic valve being the most frequently affected, closely being followed by the mitral valve. The suspicion of IE is initially evaluated with the assistance of TTE and subsequently confirmed with TOE. In terms of the pathogens involved, *Streptococcus* isolates show the highest prevalence in our review, followed by the *Staphylococcus* species. However, the prevalence of Staphylococcus remains high, and it mirrors trends seen in the US, where *Staphylococcus aureus* is often the primary culprit, trailed by *Streptococcus*. Furthermore, fungi and HACEK bacteria groups are rarely implicated in endocarditis cases, as supported by both our findings and the literature. The empirical antibiotic treatment in the studies analyzed was adjusted based on the results of blood or vegetation cultures, with antibiotics tailored to target each specific pathogen or antifungal agent.

Regarding septic embolization, the LAD has emerged as the most commonly affected coronary artery, consistent with the data we reviewed, followed by the RCA. Among the ACS, STEMI was more prevalent than NSTEMI in our study. The emergency management of ACS typically leaned towards interventional procedures like percutaneous thrombectomy, sometimes accompanied by balloon or stent placements. Additionally, complex surgical interventions such as surgical embolectomies or coronary bypasses, often coupled with valve replacements, were also observed, in line with current trends in the literature. The mortality rate in cases of endocarditis complicated by septic embolization leading to myocardial infarction is exceedingly high. Also, an early interdisciplinary heart team approach to patients with IE is very important.

We want to highlight the numerous challenges facing the healthcare system regarding the topic at hand. Additionally, we urge all medical professionals involved in managing IE to share their cases, especially the ones with unique complications like embolic-related ACS, to expand the pool of evidence available. This collaborative effort could pave the way for more informed and attentive approaches to tackling this serious condition in the future. Moreover, future studies involving larger patient cohorts are essential to assess the optimal treatment strategies for IE complicated by ACS. Implementing the recommendations derived from such studies into clinical practice guidelines for the multidisciplinary management of these patients is crucial. Ultimately, these collective endeavors are aimed at enhancing the clinical outcomes by placing the patient at the forefront of innovative medical care.

## Figures and Tables

**Table 1:** Scientific articles reviewing the current state and challenges in the diagnosis and management of infective endocarditis complicated by septic embolic-induced acute myocardial infarction.

Involved Valve	Cultures	Year of Publication	Author	Study Design	Type of AMI
IE of Prosthetic Aortic Valve	*Staphylococcus aureus*	2021	[18] Valencia, J.	Case report	STEMI
IE of Bioprosthetic Aortic Valve	*Streptococcus mitis*/*oral*	2020	[19] Cohen, A.	Case report	ACS
IE of Bioprosthesis Aortic Valve (perivalvular abscess)	*Staphylococcus aureus*	2022	[20] Denegri, A.	Case report	STEMI
IE of Prosthetic Aortic Valve	*Candida albicans*	2014	[21] Maqsood, K.	Case report	STEMI
IE of Bioprosthetic Aortic Valve (with an abscess)	*Enterococcus faecalis*	2016	[22] Inayat, F.	Case report	STEMI
IE of Bicuspid Aortic Valve	*Abiotrophia defectiva*	2020	[23] Khiatah, B.	Case report	STEMI
IE of Bicuspid Aortic Valve (root access)	*Staphylococcus species*	2012	[24] Hibbert, B.	Case report	STEMI
IE of Aortic Valve	*Candida* spp.	2020	[25] Ghazzal, A.	Case report	STEMI
IE of Aortic Valve	*Methicillin-susceptible* *Staphylococcus aureus* (MSSA)	2020	[26] Bolton, A.	Case report	STEMI
IE of Aortic Valve	*Staphylococcus* *hominins*	2023	[27] Dina, A. J.	Case report	NSTEMI
IE of Aortic Valve (pseudoaneurysm in the aortic root)	*Enterococcus faecium*	2023	[28] Molina-Lopez, V. H.	Case report	NSTEMI
IE of Aortic Valve	*Streptococcus mutans* group bacteria	2023	[29] Oomrigar, S.	Case report	STEMI
IE of Aortic Valve	*Streptococcus anginosus*	2008	[30] Christiaens, L.	Case report	ACS
IE of Aortic Valve	Negative	2022	[31] Boulouiz, S.	Case report	NSTEMI
IE of Mitral Valve	*Streptococcus salivarius*	2023	[32] Ściborski, K.	Case report	STEMI
IE of Mitral Valve	*Abiotrophia defectiva*	2023	[33] Toda, K.	Case report	STEMI
IE of Mitral Valve	Negative (Libman–Sacks endocarditis)	2023	[34] Banerjee, S.	Case report	NSTEMI
IE of Mitral Valve	Negative	2022	[35] Carvalho Gouveia, C.	Case report	STEMI
IE of Mitral Valve	MSSA	2024	[36] Massey, B. L.	Case report	ACS
IE of Mitral valve	*Listeria monocytogenes*	2020	[37] Zhao, J.	Case report	STEMI
IE of Mitral Valve	*Enterococcus faecalis*	2022	[38] Seby, R.	Case report	STEMI
IE of Mitral Valve	Negative	2020	[39] Adhikari, S.	Case report	STEMI
IE of Mitral Valve	*Streptococcus viridans*	2008	[40] Baek, M.	Case report	STEMI
IE of Mitral Valve	*Gemella*	2016	[41] Winkler, J.	Case report	STEMI
IE of Mitral Valve	*Streptococcus cristatus*	2020	[42] Liu, Y.-H.	Case report	STEMI
IE of Mitral Valve	*Cardiobacterium hominins*	2012	[43] Courand, P.-Y.	Case report	ACS
IE of Mitral Valve and Aortic Valve	*Enterococcus faecalis*	2021	[44] Usui, R.	Case report	ACS
IE of Mitral Valve and Aortic Valve	*Abiotrophia defectiva*	2022	[45] Folley, E.D.	Case report	ACS
IE of Mitral Valve and new stenosis of the recently replaced Tricuspid Valve	*Methicillin-resistant* *Staphylococcus aureus* (*MRSA*) *Streptococcus agalactiae*	2023	[46] Alla, R.	Case report	STEMI
IE of Tricuspid Valve	MSSA	2020	[47] Cohen, S.	Case report	STEMI
IE of Mitral–Aortic Junction	*Aggregatibacter aphrophilus*	2020	[48] Cardoso Monti Sousa, L. L.	Case report	STEMI

## Data Availability

Not applicable.

## Abbreviations

The abbreviations used in this article are listed below in alphabetical order.

AI	Artificial intelligence
ACS	Acute coronary syndrome
AHA	American Heart Association
AMI	Acute myocardial infarction
AMR	Antimicrobial resistance
CAD	Coronary artery disease
CAMA	Coronary artery mycotic aneurysm
CT	Computed tomography
DES	Drug-eluting stents
EE	Embolic events
ESC	European Society of Cardiology
IE	Infective endocarditis
GD	Graves’ disease
HF	Heart failure
IBD	Inflammatory bowel disease
*JACC*	*Journal of the American College of Cardiology*
IVDA	Intravenous drug abusers
LAD	Anterior descending artery
LCX	Left coronary circumflex artery
LMCA	Left main coronary artery
LSE	Libman–Sacks endocarditis
MIC	Minimum inhibitory concentration
MRI	Cardiac magnetic resonance
MRSA	*Methicillin-resistant Staphylococcus aureus*
MS	Multiple sclerosis
MSSA	*Methicillin-susceptible Staphylococcus aureus*
NSTEMI	NON-ST elevation myocardial infarction
NVE	Native valve endocarditis
PREMs	Patient-reported Experience Measures
PROMs	Patient-reported Outcome Measures
PVE	Prosthetic valve endocarditis
RA	Rheumatoid arthritis
RCA	Right coronary artery
SLE	Systemic lupus erythematosus
STEMI	ST elevation myocardial infarction
TDM	Therapeutic drug monitoring
TOE	Transesophageal cardiac ultrasound
TTE	Transthoracic cardiac ultrasound
T1D	Type 1 diabetes
T2D	Type 2 diabetes
UI	Uncontrolled infection
WHO	World Health Organization

## References

[1] Momtazmanesh S., Saeedi Moghaddam S., Malakan Rad E., Azadnajafabad S., Ebrahimi N., Mohammadi E., Rouhifard M., Rezaei N., Masinaei M., Rezaei N. (2022). Global, Regional, and National Burden and Quality of Care Index of Endocarditis: The Global Burden of Disease Study 1990–2019. Eur. J. Prev. Cardiol..

[2] Rizzo V., Salmasi M.Y., Sabetai M., Primus C., Sandoe J., Lewis M., Woldman S., Athanasiou T. (2023). Infective Endocarditis: Do We Have an Effective Risk Score Model? A Systematic Review. Front. Cardiovasc. Med..

[3] Kildahl H.A., Brenne E.L., Dalen H., Wahba A. Systemic Embolization in Infective Endocarditis. Indian J. Thorac. Cardiovasc. Surg..

[4] Amri M., Tamir E.M., Drighil A., Habbal R. (2024). An Infective Endocarditis Complicated by Multiple Septic Emboli: Case Report. Egypt. Heart J..

[5] Cabezón G., López J., Vilacosta I., Sáez C., García-Granja P.E., Olmos C., Jerónimo A., Gutiérrez Á., Pulido P., de Miguel M. (2022). Reassessment of Vegetation Size as a Sole Indication for Surgery in Left-Sided Infective Endocarditis. J. Am. Soc. Echocardiogr..

[6] Otto C.M., Nishimura R.A., Bonow R.O., Carabello B.A., Erwin J.P., Gentile F., Jneid H., Krieger E.V., Mack M., McLeod C. (2021). 2020 ACC/AHA Guideline for the Management of Patients with Valvular Heart Disease: Executive Summary. J. Am. Coll. Cardiol..

[7] Özkan U. (2024). A Novel Potential Biomarker for Predicting the Development of Septic Embolism in Patients with Infective Endocarditis: Systemic Coagulation Inflammation Index. Turk. Kardiyol. Dern. Ars..

[8] Taj S., Arshad M.U., Khan H., Sidhu G.S., Singh R. (2021). Infective Endocarditis Leading to Intracranial Abscess: A Case Report and Literature Review. Cureus.

[9] Ati N.S.D.L., Subagjo A., Muhammad R., Aditya M. (2023). Acute on Chronic Limb-Threatening Ischemia Associated with Septic Embolism in Patient with Infective Endocarditis. Pak. Heart J..

[10] Roux V., Salaun E., Tribouilloy C., Hubert S., Bohbot Y., Casalta J.-P., Barral P.-A., Rusinaru D., Gouriet F., Lavoute C. (2017). Coronary Events Complicating Infective Endocarditis. Heart.

[11] Hu W., Wang X., Su G. (2021). Infective Endocarditis Complicated by Embolic Events: Pathogenesis and Predictors. Clin. Cardiol..

[12] Habib G., Lancellotti P., Antunes M.J., Bongiorni M.G., Casalta J.-P., Zotti D. (2015). ESC Guidelines for the Management of Infective Endocarditis. Eur. Heart J..

[13] Delgado V., Ajmone Marsan N., de Waha S., Bonaros N., Brida M., Burri H., Caselli S., Doenst T., Ederhy S., Erba P.A. (2023). 2023 ESC Guidelines for the Management of Endocarditis. Eur. Heart J..

[14] McDonald E.G., Aggrey G., Tarık Aslan A., Casias M., Cortes-Penfield N., Dong M.Q.D., Egbert S., Footer B., Isler B., King M. (2023). Guidelines for Diagnosis and Management of Infective Endocarditis in Adults: A WikiGuidelines Group Consensus Statement. JAMA Netw. Open.

[15] Nazir S., Elgin E., Loynd R., Zaman M., Donato A. (2019). ST-Elevation Myocardial Infarction Associated with Infective Endocarditis. Am. J. Cardiol..

[16] Konstantinides S.V., Meyer G., Becattini C., Bueno H., Geersing G.-J., Harjola V.-P., Huisman M.V., Humbert M., Jennings C.S., Jiménez D. (2020). 2019 ESC Guidelines for the Diagnosis and Management of Acute Pulmonary Embolism Developed in Collaboration with the European Respiratory Society (ERS). Eur. Heart J..

[17] Ibanez B., James S., Agewall S., Antunes M.J., Bucciarelli-Ducci C., Bueno H., Caforio A.L.P., Crea F., Goudevenos J.A., Halvorsen S. (2018). 2017 ESC Guidelines for the Management of Acute Myocardial Infarction in Patients Presenting with ST-Segment Elevation: The Task Force for the Management of Acute Myocardial Infarction in Patients Presenting with ST-Segment Elevation of the European Society of Cardiology (ESC). Eur. Heart J..

[18] Valencia J. (2020). Aortic Prosthetic Valve Endocarditis as a Cause of Acute Myocardial Infarction. REC Interv. Cardiol. (Engl. Ed.).

[19] Cohen A., Greco J., Levitus M., Nelson M. (2020). The Use of Point-of-care Ultrasound to Diagnose Infective Endocarditis Causing an NSTEMI in a Patient with Chest Pain. J. Am. Coll. Emerg. Physicians Open.

[20] Denegri A., Venturelli A., Boriani G. (2021). Infective Endocarditis with Perivalvular Abscess Complicated by Septic Embolization with Acute ST-Segment Elevation Myocardial Infarction and Peripheral Ischemia. Int. J. Cardiol. Heart Vasc..

[21] Maqsood K., Sarwar N., Eftekhari H., Lotfi A. (2014). Septic Coronary Artery Embolism Treated with Aspiration Thrombectomy: Case Report and Review of Literature. Tex. Heart Inst. J..

[22] Inayat F., Virk H.H., Farooq S., Ghani A., Mirrani G., Athar M. (2016). Prosthetic Aortic Valve Endocarditis with Left Main Coronary Artery Embolism: A Case Report and Review of the Literature. N. Am. J. Med. Sci..

[23] Khiatah B., Jazayeri S., Wilde J., Westfall M., Kong T.Q., Frugoli A. (2020). ST-Segment Elevation Myocardial Infarction from Septic Emboli Secondary to Infective Endocarditis by Abiotrophia Defectiva. Case Rep. Cardiol..

[24] Hibbert B., Kazmi M., Veinot J.P., O’Brien E.R., Glover C. (2012). Infective Endocarditis Presenting as ST-Elevation Myocardial Infarction: An Angiographic Diagnosis. Can. J. Cardiol..

[25] Ghazzal A., Gill G.S., Radwan S., Barnett C. (2020). Embolic ST-Elevation Myocardial Infarction from Candida Endocarditis. Cureus..

[26] Bolton A., Hajj G., Payvandi L., Komanapalli C. (2020). ST-Segment Elevation Caused by Ostial Right Coronary Artery Obstruction in Infective Endocarditis: A Case Report. BMC Cardiovasc. Disord..

[27] Dina A.J., Hicks S., Khoury C. (2023). Infective Endocarditis Presenting with Non-ST Elevation Myocardial Infarction: A Case Report. Cureus.

[28] Molina-Lopez V.H., Diaz-Rodriguez P.E., Rivera-Torres J.J., Vazquez-Fuster J., Maldonado-Suarez J., Vicenty-Rivera S. (2023). Unusual Case of Acute Coronary Syndrome Due to Compression of the Left Main Coronary Artery from a Contained Aortic Root Perforation. Cureus.

[29] Oomrigar S., Rojas Rodriguez R., Macias T., Bonilla D. (2023). The Emboli Does Not Fall Far from the Vegetation: A Case of Infective Endocarditis Causing Acute Myocardial Infarction. Chest.

[30] Christiaens L., Mergy J., Franco S., Serrano L., Ardilouze P. (2008). Aortic Valvular Endocarditis with Mobile Vegetations and Intracoronary Embolism: Demonstration by Cardiac Multislice Computed Tomography. Eur. Heart J..

[31] Boulouiz S., Bouchlarhem A., Amaqdouf S., Noha E.O., Bazid Z. (2022). Acute Coronary Syndrome Complicating Infective Endocarditis: A Case Report with an Etiological Review. Ann. Med. Surg..

[32] Ściborski K., Stopyra-Początek M., Szczepański A., Furdal M., Gać P., Pieróg M., Telichowski A., Banasiak W. (2023). Giant Mycotic Aneurysms of the Coronary Artery after Stent Implantation for Myocardial Infarction Due to Infective Endocarditis. Kardiol. Pol..

[33] Toda K., Takagi K., Noguchi T. (2023). Silent Rapid Progression of Mycotic Left Main Aneurysm Following Stenting to Bailout for Acute Occlusion Caused by Infective Endocarditis. Eur. Heart J..

[34] Banerjee S., Ahmed M., Osei-Sarpong J., Vasigh M., Aiello D. (2023). Libman-Sacks Endocarditis Presenting as Acute Coronary Syndrome, Acute Heart Failure and Multiple Embolic Strokes. Cureus.

[35] Carvalho Gouveia C., Pimenta I., Fernandes M., Chambino B., Côrte-Real H. (2022). Mitral Valve Infective Endocarditis Complicated with Coronary Artery Embolization. Cureus.

[36] Massey B.L., Lee K., Lei K., Al-Taweel O., Ciuffo G., Ahsan C.H. (2024). Mitral Valve Endocarditis Complicated by Septic Coronary Embolism Resulting in Ventricular Septal Rupture. J. Am. Coll. Cardiol..

[37] Zhao J., Yang J., Chen W., Yang X., Liu Y., Cong X., Huang Z., Li N. (2020). Acute Myocardial Infarction as the First Sign of Infective Endocarditis: A Case Report. J. Int. Med. Res..

[38] Seby R., Kim C., Khreis M., Khreis K. (2022). *Enterococcus faecalis*-Induced Infective Endocarditis: An Unusual Source of Infection and a Rare Clinical Presentation. J. Int. Med. Res..

[39] Adhikari S., Gajurel R.M., Paudel C.M., Devkota S., Shakya S., Koirala P., Thapa S., Pathak S.R., Sharma M., Yadav V. (2020). An Extraordinary Case of Infective Endocarditis at Its Extreme Forms of Systemic Embolisation: A Rare Case Report. Nepal. Heart J..

[40] Baek M., Kim H., Yu C., Na C. (2008). Mitral Valve Surgery with Surgical Embolectomy for Mitral Valve Endocarditis Complicated by Septic Coronary Embolism. Eur. J. Cardiothorac. Surg..

[41] Winkler J., Chaudhry S.-P., Stockwell P.H. (2016). Gemella Endocarditis Presenting as an ST-Segment-Elevation Myocardial Infarction. Tex. Heart Inst. J..

[42] Liu Y.-H., Lee W.-H., Chu C.-Y., Su H.-M., Lin T.-H., Yen H.-Y., Voon W.-C., Lai W.-T., Sheu S.-H., Hsu P.-C. (2018). Infective Endocarditis Complicated with Nonobstructive ST Elevation Myocardial Infarction Related to Septic Embolism with Intracranial Hemorrhage: A Case Report. Medicine.

[43] Courand P.-Y., Mouly-Bertin C., Thomson V., Lantelme P. (2012). Acute Coronary Syndrome Revealed Cardiobacterium Hominis Endocarditis. J. Cardiovasc. Med..

[44] Usui R., Mutsuga M., Narita Y., Tokuda Y., Terazawa S., Ito H., Uchida W., Usui A. (2021). Sudden Circulatory Collapse Caused by Mechanical Obstruction of the Left Main Coronary Trunk with Infective Endocarditis Vegetation: A Case Report. Surg. Case Rep..

[45] Foley E.D., Ben Omran M., Bora V., Castresana M.R. (2018). Cardiogenic and Septic Shock Associated with Aortic and Mitral Valve Infective Endocarditis Caused by *Abiotrophia Defectiva* from a Urinary Tract Infection. SAGE Open Med. Case Rep..

[46] Alla R., Lopez J., Dutta M., Patel N., Taha Y. (2023). Abstract 17621: Acute Coronary Syndrome Due to Septic Emboli from Infective Endocarditis. Circulation.

[47] Cohen S., Ford L., Situ-LaCasse E., Tolby N. (2020). Infective Endocarditis Causing Acute Myocardial Infarction. Cureus.

[48] Cardoso Monti Sousa L.L., de Carvalho Andreucci Torres Leal T., de Matos Soeiro A., Soares P.R., Scudeler T.L. (2020). Septic Coronary Embolism Treated with Manual Aspiration Thrombectomy: A Case Report. Med. Case Rep. Study Protoc..

[49] Zoghbi W.A., Jone P.-N., Chamsi-Pasha M.A., Chen T., Collins K.A., Desai M.Y., Grayburn P., Groves D.W., Hahn R.T., Little S.H. (2024). Guidelines for the Evaluation of Prosthetic Valve Function with Cardiovascular Imaging: A Report from the American Society of Echocardiography Developed in Collaboration with the Society for Cardiovascular Magnetic Resonance and the Society of Cardiovascular Computed Tomography. J. Am. Soc. Echocardiogr..

[50] Baddour L.M., Wilson W.R., Bayer A.S., Fowler V.G., Tleyjeh I.M., Rybak M.J., Barsic B., Lockhart P.B., Gewitz M.H., Levison M.E. (2015). Infective Endocarditis in Adults: Diagnosis, Antimicrobial Therapy, and Management of Complications: A Scientific Statement for Healthcare Professionals from the American Heart Association. Circulation.

[51] Thygesen K., Alpert J.S., Jaffe A.S., Chaitman B.R., Bax J.J., Morrow D.A., White H.D., Thygesen K., Alpert J.S., Jaffe A.S. (2019). Fourth Universal Definition of Myocardial Infarction (2018). Eur. Heart J..

[52] Acar Z. (2020). An Unusual Cause of Acute Coronary Syndrome: Left Ventricular Outflow Tract Pseudoaneurysm. Anatol. J. Cardiol..

[53] Hali R., Sharifkazemi M., Yaminisharif A., Bagheri J., Shahbazi N. (2023). Coronary Artery Mycotic Aneurysm in a Patient Suffering from Subacute Endocarditis: A Case Report and Literature Review. Front. Cardiovasc. Med..

[54] Tagliari F., Ribeiro C.L., de Carvalho G.P.V., Tagliari L.P., Weksler C., Lamas C. (2020). Acute Coronary Syndrome, a Rare Manifestation of Infective Endocarditis: A Case Report. Heart Vessel. Transpl..

[55] Sharma D., Sulaiman Z.I., Tu P.J., Harrell S., Cavalieri S., Skidmore P.J., Baer S.L. (2024). A Case of Infective Endocarditis Caused by *Citrobacter Koseri*: Unraveling a Rare Pathogen and Dire Outcome. J. Investig. Med. High. Impact Case Rep..

[56] Rudrappa M., Kokatnur L. (2017). Infective Endocarditis Due to Abiotrophia Defectiva and Its Feared Complications in an Immunocompetent Person: Rare, but Real. J. Glob. Infect. Dis..

[57] Yucel E., Bearnot B., Paras M.L., Zern E.K., Dudzinski D.M., Soong C.-P., Jassar A.S., Rosenfield K., Lira J., Lambert E. (2022). Diagnosis and Management of Infective Endocarditis in People Who Inject Drugs. J. Am. Coll. Cardiol..

[58] Hermanns H., Alberts T., Preckel B., Strypet M., Eberl S. (2023). Perioperative Complications in Infective Endocarditis. J. Clin. Med..

[59] Calero-Núñez S., Ferrer Bleda V., Corbí-Pascual M., Córdoba-Soriano J.G., Fuentes-Manso R., Tercero-Martínez A., Jiménez-Mazuecos J., Barrionuevo Sánchez M.I. (2018). Myocardial Infarction Associated with Infective Endocarditis: A Case Series. Eur. Heart J. Case Rep..

[60] Siquier-Padilla J., Cuervo G., Urra X., Quintana E., Hernández-Meneses M., Sandoval E., Lapeña P., Falces C., Mestres C.A., Paez-Carpio A. (2022). Optimal Timing for Cardiac Surgery in Infective Endocarditis with Neurological Complications: A Narrative Review. J. Clin. Med..

[61] Hill T.E., Kiehl E.L., Shrestha N.K., Gordon S.M., Pettersson G.B., Mohan C., Hussein A., Hussain S., Wazni O., Wilkoff B.L. (2021). Predictors of Permanent Pacemaker Requirement after Cardiac Surgery for Infective Endocarditis. Eur. Heart J. Acute Cardiovasc. Care.

[62] Eranki A., Wilson-Smith A.R., Ali U., Saxena A., Slimani E. (2021). Outcomes of Surgically Treated Infective Endocarditis in a Western Australian Population. J. Cardiothorac. Surg..

[63] Kashani H.H., Mosienko L., Grocott B.B., Glezerson B.A., Grocott H.P. (2020). Postcardiac Surgery Acute Stroke Therapies: A Systematic Review. J. Cardiothorac. Vasc. Anesth..

[64] Ali J.M., Gerrard C., Clayton J., Moorjani N. (2019). Reduced Re-Exploration and Blood Product Transfusion after the Introduction of the Papworth Haemostasis Checklistdagger. Eur. J. Cardiothorac. Surg..

[65] Yu Z., Fan B., Wu H., Wang X., Li C., Xu R., Su Y., Ge J. (2016). Multiple Systemic Embolism in Infective Endocarditis Underlying in Barlow’s Disease. BMC Infect. Dis..

[66] Laguna G., Blanco M., Fernández-Collantes Á., Carrascal Y. (2017). Isolated Infective Endocarditis of the Left Ventricular Outflow Tract and Multiple Septic Embolisms. Ann. Thorac. Surg..

[67] Robson C., Tan B., Stuart R., Nicholls S., Rogers B.A., Sandaradura I. (2023). A systematic review of optimal pharmacokinetic/pharmacodynamic parameters for beta-lactam therapy in infective endocarditis. J. Antimicrob. Chemother..

[68] Rybak M.J., Le J., Lodise T.P., Levine D.P., Bradley J.S., Liu C., Mueller B.A., Pai M.P., Wong-Beringer A., Rotschafer J.C. (2020). Therapeutic Monitoring of Vancomycin for Serious Methicillin-Resistant *Staphylococcus aureus* Infections: A Revised Consensus Guideline and Review by the American Society of Health-System Pharmacists, the Infectious Diseases Society of America, the Pediatric Infectious Diseases Society, and the Society of Infectious Diseases Pharmacists. Am. J. Health Syst. Pharm..

[69] Dorel M., Albert R., Le Bot A., Caillault L., Lalanne S., Tattevin P., Verdier M.-C., Lemaignen A., Revest M. (2023). Amoxicillin Therapeutic Drug Monitoring for Endocarditis: A Comparative Study (EI-STAB). Int. J. Antimicrob. Agents.

[70] Macheda G., El Helali N., Péan de Ponfilly G., Kloeckner M., Garçon P., Maillet M., Tolsma V., Mory C., Le Monnier A., Pilmis B. (2022). Impact of Therapeutic Drug Monitoring of Antibiotics in the Management of Infective Endocarditis. Eur. J. Clin. Microbiol. Infect. Dis..

[71] Rybak M.J., Lomaestro B.M., Rotschafer J.C., Moellering R., Craig W., Billeter M., Dalovisio J.R., Levine D.P. (2009). Therapeutic monitoring of vancomycin in adult patients: A consensus review of the American Society of Health-System Pharmacists, the Infectious Diseases Society of America, and the Society of Infectious Diseases Pharmacists. Am. J. Health-Syst. Pharm..

[72] Cairns K.A., Abbott I.J., Dooley M.J., Peleg A.Y., Peel T.N., Udy A.A. (2023). The Impact of Daptomycin Therapeutic Drug Monitoring on Clinical Outcomes: A Systematic Review. Int. J. Antimicrob. Agents.

[73] Gallerani A., Gatti M., Bedini A., Casolari S., Orlando G., Puzzolante C., Franceschini E., Menozzi M., Santoro A., Barp N. (2023). Long-Term Suppressive Therapeutic-Drug-Monitoring-Guided Dalbavancin Therapy for Cardiovascular Prosthetic Infections. Antibiotics.

[74] Stefanache A., Lungu I.-I., Butnariu I.-A., Calin G., Gutu C., Marcu C., Grierosu C., Bogdan Goroftei E.R., Duceac L.-D., Dabija M.G. (2023). Understanding How Minerals Contribute to Optimal Immune Function. J. Immunol. Res..

[75] French R., Boccelli A., Valosky K., Oliver E., Uritsky T., McCullion J., Zwiebel S., Andrews T. (2024). A Promising Approach to Addressing the Needs of Patients with Endocarditis Secondary to Injection Drug Use: A Case Report. Health Soc. Work..

[76] Kyaw M., Aye T. (2023). 33 Heart, and Thyroid: A Story of Partners in Crime. ACHD/Valve Dis./Pericard. Dis./Cardiomyopathy.

[77] Bumbacea R.S., Popa L.G., Orzan O.A., Voiculescu V.M., Giurcaneanu C. (2014). Clinical and Therapeutic Implications of the Association between Chronic Urticaria and Autoimmune Thyroiditis. Acta Endocrinol..

[78] Stewart M., Brown J.B., Wayne Weston W., Freeman T., Ryan B.L., McWilliam C.L., McWhinney I.R. (2024). Patient-Centered Medicine: Transforming the Clinical Method.

[79] Fontaine G., Poitras M.-E., Sasseville M., Pomey M.-P., Ouellet J., Brahim L.O., Wasserman S., Bergeron F., Lambert S.D. (2024). Barriers and Enablers to the Implementation of Patient-Reported Outcome and Experience Measures (PROMs/PREMs): Protocol for an Umbrella Review. Syst. Rev..

[80] Harrison R., Iqbal M.P., Chitkara U., Adams C., Chauhan A., Mitchell R., Manias E., Alston M., Hadley A.M. (2024). Approaches for Enhancing Patient-Reported Experience Measurement with Ethnically Diverse Communities: A Rapid Evidence Synthesis. Int. J. Equity Health.

[81] Rappold D., Stättner S., Nöhammer E. (2024). Patient-Reported Outcome and Experience Measures (PROM/PREM) in Patients Undergoing Liver Surgery with Enhanced Recovery after Surgery (ERAS^®^): An Exploratory Study. Healthcare.

[82] Pentescu A., Orzan M.C., Stefanescu C.D., Orzan O.A. (2014). Modelling Patient Satisfaction in Healthcare. J. Econ. Comput. Econ. Cybern. Stud. Res..

[83] Lai C.K.-C., Leung E., He Y., Ching-Chun C., Oliver M.O.Y., Qinze Y., Li T.C.-M., Lee A.L.-H., Li Y., Lui G.C.-Y. (2024). A Machine Learning-–Based Risk Score for Prediction of Infective Endocarditis among Patients with *Staphylococcus aureus* Bacteremia—The SABIER Score. J. Infect. Dis..

[84] Galizzi Fae I., Murta Pinto P.H.O., De Oliveira G.B., Taconeli C.A., De Andrade A.B., De Padua L.B., Diamante L.C., Ferrari T.C.A., Nunes M.C.P. (2023). Cardiac Complications as a Major Predictor of In-Hospital Death in Infective Endocarditis Using Machine-Learning Algorithm Analysis. Eur. Heart J..

[85] Yi C., Zhang H., Yang J., Chen D., Jiang S. (2024). Elucidating Common Pathogenic Transcriptional Networks in Infective Endocarditis and Sepsis: Integrated Insights from Biomarker Discovery and Single-Cell RNA Sequencing. Front. Immunol..

